# Supplementary material for: Circulating causal protein networks linked to future risk of myocardial infarction
Source: Nat Commun. 2025 Dec 18;17:448. doi: 10.1038/s41467-025-67135-3 (PMC12800284; doi:10.1038/s41467-025-67135-3)
Supplement: Supplementary file 1 — Supplementary Information [file 41467_2025_67135_MOESM1_ESM.pdf]

SUPPLEMENTARY INFORMATION

Circulating causal protein networks linked to future risk of myocardial infarction

Sean Bankier[1,2,*], Valborg Gudmundsdottir[2,3,*], Thorarinn Jonmundsson[2], Heida Bjarnadottir[2], Joseph Loureiro[4], Lingfei Wang[5], Elísabet A. Frick[2], Nancy Finkel[4], Anthony P Orth[6], Thor Aspelund[2,3], Lenore J Launer[7], Johan LM Björkegren[8], Lori L Jennings[4], John R Lamb[9], Vilmundur Gudnason[2,3,†], Tom Michoel[1,†] and Valur Emilsson[2,3,†]

[1]Computational Biology Unit, Department of Informatics, University of Bergen, P.O. Box 7803, 5020 Bergen, Norway
[2]Icelandic Heart Association, Holtasmari 1, IS-201 Kopavogur, Iceland.
[3]Faculty of Medicine, University of Iceland, 101 Reykjavik, Iceland.
[4]Novartis Biomedical Research, 22 Windsor Street, Cambridge, MA 02139, USA
[5]University of Massachusetts Chan Medical School. Worcester MA 01605, USA
[6]Novartis Biomedical Research, 10675 John Jay Hopkins Drive, San Diego, CA 92121, USA
[7]Laboratory of Epidemiology and Population Sciences, National Institute on Aging, MD, USA
[8]Department of Medicine, Karolinska Institutet, Karolinska Universitetssjukhuset, Huddinge, Sweden
[9]Monoceros Biosystems, 12636 High Bluff Drive, Suite 400, San Diego, CA. 92130, USA

*These authors contributed equally
†These authors jointly supervised this work: valur@hjarta.is, tom.michoel@uib.no and v.gudnason@hjarta.is.

Supplementary Text

Variance explained by parent nodes

Having identified independent *cis*-acting protein SNPs for every network regulator, we estimated the proportion of variance in protein expression that could be explained by *cis*-acting pQTLs using multiple linear regression. For each protein we fitted a linear model in R (version 4.3.2), where the genotypes for the independent *cis*-acting pSNPs act as the explanatory variables for protein expression. The adjusted coefficient of determination (adjusted r^2) from this model was used as an estimate of the variance explained by the *cis* component for each protein, and the mean adjusted r^2 was then calculated across all network regulators.

We also estimated the variance in target protein expression that could be explained by *cis*-acting pQTLs from the target regulators. For every target protein, we fitted a linear model, as described previously, using the genotypes of all independent *cis*-acting pQTLs for the regulators (parental nodes), in addition to any *cis*-acting pQTLs for the target itself. We then calculated the difference in variance explained by local and parental *cis*-acting pQTLs combined, to that explained by *cis*-acting genetic variation alone. If the target protein did not have any *cis*-acting pQTLs, then the variance explained by the *cis* component was set to 0. Any unresolved networks were excluded from this analysis.

We examined the impact that *cis*-acting pQTLs for network regulators (parental nodes) had upon the expression of 5,459 target proteins in the CPN, including 162 target proteins that were also regulators for other proteins. For each target protein, we estimated the proportion of variance in protein expression (adjusted r^2) that could be explained by *cis*-acting pQTLs for all regulatory proteins, in addition to any independent *cis*-acting pQTLs for this protein. We found that the number of regulators a target protein has is correlated with how much variance can be explained by those regulators (Spearman R = 0.78) (Supplementary Fig. S3A-B). We observed 32 cases

where more than 50% of the variance in the expression of the target protein is explained by the *cis*-acting pQTLs of the parents alone, with no local *cis*-component contributing. Among these, 11 have less than 10 regulators. For example, DAP has 3 regulators: TXNRD3, KNG1 and HRG. KNG1 and HRG are within 1 KB on of each other on chromosome 3, and share 5 targets (20 targets each), however interestingly their instruments are not in LD ($R^2 < 0.2$). TXNRD3 is also on chromosome 3, but ~60 MB upstream. However, DAP is on chromosome 5 and together these *cis*-pSNPs explain 82% of the variance in DAP protein expression, with no *cis*-acting signal for DAP being observed.

Comparing the CPN with protein-protein interaction networks

We used the human integrated protein-protein interaction reference database (HIPPIE)[1] to identify experimentally derived protein-protein interactions (PPIs) that have been captured by the serum CPN described in this study. We accessed all 289,112 PPIs from the HIPPIE database, which have been scored as a weighted sum, based on the reliability of the evidence underpinning the interactions and the number of studies detecting each interaction. There are 262,346 PPIs scored at medium confidence (confidence score > 0.63) and 77,630 scored at high confidence (confidence score > 0.72), based on thresholds defined by HIPPIE authors. We then calculated the overlap between edges in the CPN with PPIs from HIPPIE at the different confidence thresholds. After which, we compared the number of common interactions between HIPPIE and the CPN to common interactions between HIPPIE and the edges from random networks. These networks were generated by randomly sampling proteins from the complete set of measured AGES proteins, to produce the same number of edges as in the CPN. This process was performed 10 times and the mean number of edges captured, in addition to the standard deviation, was calculated at each of the different confidence thresholds.

We identified edges in the serum protein network (FDR = 1%) prior to LD resolution, that had previously been identified as PPIs using the HIPPIE database. PPIs from HIPPIE have been

scored based on the strength of the supporting experimental evidence and the number of studies where and interaction has been detected. We identified 506 CPN edges that were also PPIs in HIPPIE at any score, 429 at medium confidence and 216 at high confidence, using confidence thresholds that have been defined by the HIPPIE authors (Supplementary Fig. S7). We then compared the overlap between HIPPIE and the CPN edges to the mean number of edges captured by networks generated through random sampling (Methods). We found that across all confidence thresholds, the true CPN consistently captured more edges than the mean captured by random networks: 332 edges at any score ($z = 14.6$, P-value < 0.001), 296 at medium confidence ($z = 10.3$, P-value < 0.001), and 94 at high confidence (z-score = 24.3, P-value <0.001) (Methods, Supplementary Fig. S7). It should be noted that HIPPIE measures direct PPIs and does not account for indirect interaction between proteins, potentially mediated through cross-tissue signaling which are captured by the CPN. Therefore, it is only possible to validate direct PPIs, most of which have been identified *in vitro,* and not within the context of human serum.

The causal protein networks coincide with the co-regulatory network in serum

We compared the top ranked CPN subnetworks (Table 2, main text), with the previously published serum protein co-regulatory network from the AGES study[2], focusing solely on the network regulators and corresponding target proteins detected by both the 5K and 7K platforms, as the serum co-regulatory network was reconstructed using the 5K aptamer-based platform. We observed a significant overlap between protein clusters in the two network types (Supplementary Data 9 and Supplementary Fig. S10A-D). This overlap is evident in two ways: first, many CPNs share the same co-regulatory modules, and second, when a single CPN intersects with multiple co-regulatory modules, these modules frequently belong to the same super-cluster of correlated co-regulatory modules[2]. For example, the PCDH8 CPN subnetwork overlapped with the co-regulatory modules PM16 and PM17 (Supplementary Fig. S10A), both in supercluster IV, which is strongly linked to CAD, HF, metabolic syndrome, adiposity, and overall survival[2]. AIPL1 CPN

overlapped with PM6, PM7, and PM9 (Supplementary Data 9, Supplementary Fig. S10B), all within supercluster II, that is associated with inflammation, CAD, HF, and survival[2]. Additionally, both the C2 and CFB CPNs significantly overlap with PM13 and PM15 from supercluster III (Supplementary Data 9, Supplementary Fig. S10C), which has been linked to age-related macular degeneration in the AGES study[3]. Finally, the NUDT21 CPN significantly overlaps with the PM26 and PM27 modules from supercluster V (Supplementary Data 9, Supplementary Fig. S10D), which have been previously linked to cardiovascular and metabolic diseases, as well as overall and disease-specific survival[2]. GABARAP, IZUMO1, FABP3, DCTN2, MRRF, NUDT21, and COL28A1 CPN subnetworks show significant overlaps with the large co-regulatory module PM26 (Supplementary Data 9), which contains 390 proteins, indicating that different CPNs converge into a single, large co-regulatory network. Among the 27 previously identified serum protein co-regulatory subnetworks[2], 12 did not overlap with any of the top ranked CPN subnetworks. Finally, in most instances, when the network regulator aptamer of a specific CPN was available on the 5K platform and assigned to a co-regulatory module, it was part of the overlapping co-regulatory module or super-cluster (Supplementary Data 9), suggesting that these two types of networks may share underlying genetic influences. Overall, there is a significant relationship between the circulating CPN and the co-regulatory networks, despite fundamental differences in the methodologies used for their reconstruction.

Additional links between the top-ranked networks and ACVD

Numerous pathways were enriched among the network regulators from the top ranked subnetworks listed in Table 2 (see also Supplementary Fig. S12). These include pathways previously associated with CVD pathophysiology, such as cellular heat acclimation[4], granulocyte colony-stimulating factor receptor binding[5], farnesylated protein binding[6], vitamin E binding[7] and complement system-related functions[8], among others. Functional enrichment analysis of the CPN target members within the top-ranked networks identified numerous pathways that were

over-represented across various subnetworks (Supplementary Data 11, Supplementary Fig. S13). While these pathways differ from those identified for the combined group of network regulators, both sets share enrichment in cellular response to heat shock. Finally, we explored known functional and physical protein-protein interactions among the top ranked serum protein network regulators using the STRING database[9] and observed a significant enrichment in interaction levels ($P = 0.0473$), indicating that these network regulators interact more frequently than expected by chance. This analysis reflects some of the previously identified interactions shown in Fig. 6C (main text) and offers additional insights, such as interactions involving the top ranked network regulator ITIH3, which shows physical connections with KLKB1, APOA5, and AFM (Supplementary Fig. S14).

Several of the top-ranked network regulators have been previously associated with atherosclerotic cardiovascular disease (ACVD)-related traits, including the proinflammatory protein ITIH3, which emerged as the leading subnetwork (Table 2, main text). The ITIH3 subnetwork comprises 11 protein members and is enriched in the ubiquitin-mediated proteolysis pathway (Supplementary Data 11). Both the network regulator and the corresponding eigenprotein were associated with incident MI and related traits, except for T2D (Table 2, Fig. 4, main text). Interestingly, the data-driven reconstruction of the CPN reveals that the roots of the global network regulating a diverse array of network targets converge on ITIH3 at the base of the network (Fig. 3, main text), suggesting its involvement in many regulatory pathways. Notably, a gain-of-function genetic variant in the *ITIH3* gene has previously been linked to MI and the protein is highly expressed in vascular smooth muscle cells and macrophages within human atherosclerotic lesions[10]. Other network regulators from the top-ranked subnetworks with additional links to ACVD-related traits include: 1) <u>KEAP1</u>: KEAP1 plays a crucial role in cardiovascular health by regulating the NRF2 pathway[11], which is vital for maintaining oxidative balance and protection against vascular and myocardial damage. Specifically, cardiomyocyte-

specific knockout of *Keap1* leads to NRF2 upregulation and protection against induced cardiomyocyte death and cardiac dysfunction[12]. This contrasts with the potentially protective role of circulating KEAP1 observed in our study (Supplementary Data 15). Several factors may explain this discrepancy, including differences between tissue-specific and circulating biology, where serum KEAP1 might reflect systemic processes independent of NRF2 inhibition. Alternatively, elevated circulating KEAP1 could represent a feedback response to increased NRF2 activity, serving as an indirect marker of activated antioxidant pathways. Furthermore, genetic variant across the *KEAP1* gene, show strong genetic links to low-density lipoprotein (LDL) cholesterol levels[13], as well as familial hypercholesterolemia and ischemic heart disease[14]. 2) <u>C2</u>: A human study found that C2 deficiency is linked to a higher risk of atherosclerosis and related vascular complications[15], indicating that C2 exerts a protective effect on cardiovascular health. Our finding that higher circulating C2 levels associated with increased incident MI risk may reflect differences between lifelong deficiency versus elevated protein levels in plasma. Elevated C2 could indicate heightened complement activation or inflammation contributing to acute events like MI, whereas chronic deficiency reflects impaired immune function and vascular damage. Thus, the role of C2 in cardiovascular disease may be context- and stage-dependent. Furthermore, genetic risk variants in the *C2* gene are associated with CAD[16]. 3) <u>GABARAP</u>: The gene encoding GABARAP is proximal to genetic risk variants increasing risk of hypertension and CVD (excluding rheumatic disease)[14]. 4) <u>KLKB1</u>: We recently demonstrated *via* MR analysis that KLKB1 is causally protective against calcific aortic valve disease[17], a leading cause of heart failure through aortic stenosis[18]. More specifically, higher KLKB1 levels were associated with a reduced risk of CAVD[17]. Further, genetic variants near the *KLKB1* gene, are linked to venous thromboembolism[14]. 5) <u>APOA5</u>: Mouse studies have shown that overexpression of APOA5 reduces triglyceride levels, whereas *Apoa5* knockout mice exhibit a fourfold increase in plasma triglyceride levels[19]. Additionally, variants within the gene encoding *APOA5*, are associated with coronary atherosclerosis and coronary revascularization[14], as well as hyperlipoproteinemia in multiple

studies[20]. In line with the mouse knock-out study, APOA5 demonstrated a robust protective relationship with both MI and MetS in the present study, supported by results from both colocalization and MR analyses (Supplementary Data 13-14). 6) PTPN11: Gain-of-function mutations in PTPN11 can cause Noonan syndrome[21], which is commonly linked to heart defects that may progress to heart failure, highlighting the role of PTPN11 in cardiac development and function. This effect aligns with the positive associations observed between PTPN11 and ACVD traits and poor survival in the present study (Supplementary Data 15). Furthermore, the gene encoding *PTPN11*, is linked to major coronary heart disease events[14], and right ventricular end-diastolic volume in HF[22]. 7) CFB: Consistent with the findings of the present study, CFB has been positively associated with several ACVD-related traits in humans[23], whereas *Cfb* knockout rats display improved glucose metabolism and cardiovascular profiles in a spontaneously hypertensive rat model[23]. Additionally, variants across the gene *CFB* are linked to risk of CAD[24]. 8) C11orf49 and LRP4: the colocalized genes (defined as single regulator) on chromosome 11 encoding C11orf49 or LRP4, are associated with cardiomyopathy[14] or venous thromboembolism[25], respectively. Interestingly, both C11orf49 and LRP4 were supported as having a causal relationship with MI in this study, based on findings from both the MR and colocalization analyses (Supplementary Data 13-14). However, the direction of effect is inconsistent between the causal inference tests and their associations with ACVD-related traits (Supplementary Data 15). Neither of these proteins have previously been associated with ACVD in other studies. 9) HSPA1A and HSPA1B: The genes encoding HSPA1A and HSPA1B are tandemly located on chromosome 6p21.33 in the MHC class III region. Previously, HSPA1A and HSPA1B were causally linked to T2D and its microvascular complications through both MR and colocalization analyses[26]. In our study, HSPA1A was supported as being causally related to MetS, as indicated by both MR and colocalization analyses (Supplementary Data 13-14). It is of note that although *HSPA1A* and *HSPA1B* genes encode highly similar Hsp70 proteins, they differ in their regulation, especially in how and when they are expressed[27]. This has implications for

interpreting genetic associations, expression dynamics, and protein-level findings for these related proteins. In fact, In our study, HSPA1A, but not HSPA1B, was supported of being causally related to MetS, suggesting potential functional differences despite their high sequence similarity. 10) COL28A1: In a sub study of the large EXSCEL clinical trial involving individuals with T2D, COL28A1 levels were positively associated with HF and its key subtypes[28], indicating a possible involvement of this collagen protein in HF pathogenesis. These findings align with the present study, which demonstrates that elevated COL28A1 levels are linked to a higher risk of MI and worse survival outcomes (Supplementary Data 15). 11) CSF3: Cardiac myocytes express CSF3 under both normal and stress conditions, especially following ischemia or injury, which contributes to the promotion of their survival after a heart attack[5]. This contrasts with the present finding of a positive association between circulating CSF3, incident MI, and reduced survival (Supplementary Data 15), which may reflect a compensatory response to early cardiac injury, with elevated CSF3 acting as a biomarker of endogenous protective mechanisms. 12) FABP3: The network regulator FABP3 (heart-type) is highly expressed in the heart and rises early during myocardial injury[29]. Moreover, while *Fabp3* overexpression in mice exacerbates cardiac dysfunction after MI, its knockdown improves myocardial structural remodeling[30]. This aligns with the positive association of the regulator with ACVD-related traits observed in our study (Supplementary Data 15). Several of the top-ranked network regulators, including for instance DDX39B, have not been previously linked directly to ACVD-related traits. In our MR and colocalization analyses, DDX39B supported having causal relationship with T2D and MetS (Supplementary Data 13). The regulator protein DDX39B has been shown to play a role in modulating the NF-κB response[31], a key pathway involved in immune and inflammatory responses. This modulation may have implications for diseases like atherosclerosis and diabetes, where NF-κB signaling is frequently dysregulated causing inflammation in the vascular wall, insulin resistance and/or beta-cell dysfunction[32].

Therapeutic pathways and clinical implications

We evaluated whether the network regulators are druggable or targeted by existing compounds using public databases. Among the 25 network regulators, 13 are considered druggable via small molecules or biologics, and 6 have licensed or investigational drugs currently targeting them (Supplementary Data 16). The causal network analysis identified interconnected networks and therapeutic pathways that could be exploited for early ASCVD detection and prevention, supporting both single- and multi-pathway targeting approaches: Complement-mediated inflammation pathway: C2 and CFB are central to the alternative complement pathway, with CFB targeted by the antisense oligonucleotide Sefaxersen (RO743466), currently in Phase III trials for IgA nephropathy (Supplementary Data 16). These proteins drive vascular inflammation and atherosclerosis[33], suggesting that targeting this pathway could prevent early inflammatory progression in atherosclerotic lesions. Cellular stress response and cytoprotective pathways: KEAP1 regulates the Nrf2 oxidative stress response[12], while HSPA1A and HSPA1B control protein folding under cellular stress[34]. All three proteins have licensed drugs targeting them. Targeting these pathways may protect against oxidative damage and endothelial dysfunction, key early events in atherosclerosis. Hemostatic balance and thrombosis prevention: The key target KLKB1, a central regulator of plasma kallikrein activity in the intrinsic coagulation pathway[35], is targeted by the approved inhibitor Berotralstat against angioedema (Supplementary Data 16), highlighting a potential strategy to prevent dysregulated coagulation that contributes to thrombotic complications in ASCVD. However, our integrative analysis suggests that activation of KLKB1 may represent a potential early intervention that may reduce progression from stable to unstable disease (see Table 3, main text). Lipid metabolism and transport: The network regulators APOA5 and AFM control triglyceride-rich lipoprotein metabolism, lipid transport, and oxidative stress protection[19,36,37]. Both are classified as bio-druggable (Supplementary Data 16) and could be targeted to prevent dyslipidemia-driven initiation of atherosclerosis. Our network-based framework provides a roadmap for next-generation ASCVD prevention strategies that focus on

causal biological mechanisms, with concordant data strengthening target confidence and inconsistencies pointing to opportunities for novel therapeutic discovery.

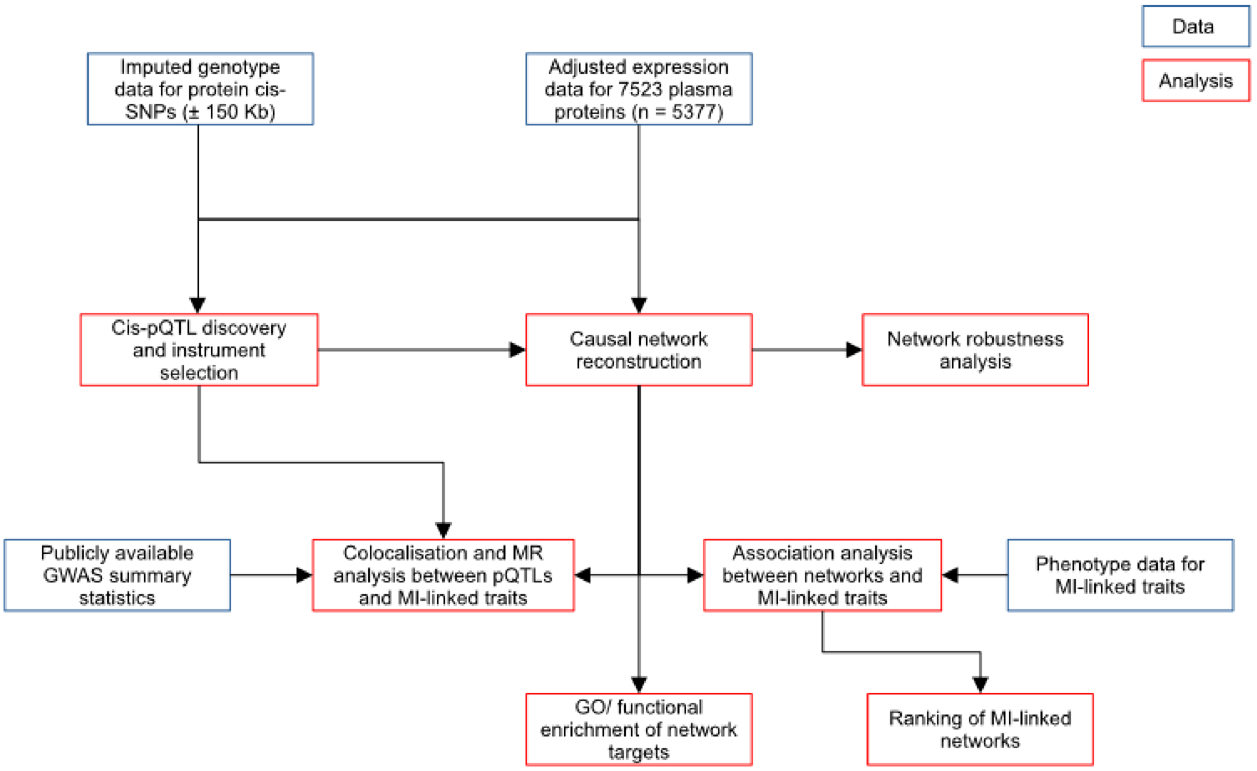

Supplementary Fig. S1. A flowchart illustrating the reconstruction and analysis of the circulating causal protein networks (CPNs) in relation to incident myocardial infarction (MI) and associated phenotypes. An alternate version of the study overview is presented in Fig. 1 of the main text.

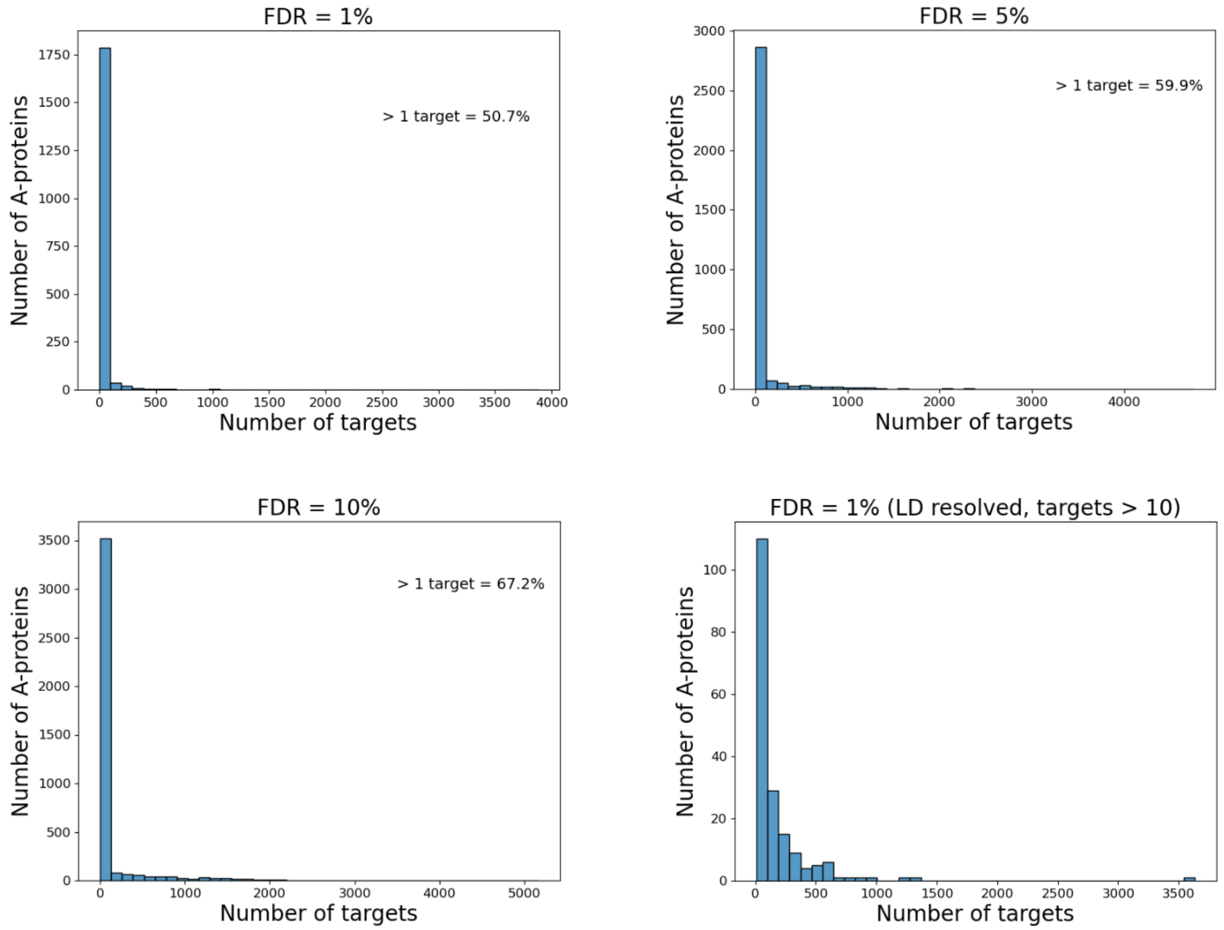

Supplementary Fig. S2. Histograms of network target distributions are shown at various FDR thresholds, followed by the target distribution in the CPN based on a minimum of 10 unique protein members per subnetwork.

A

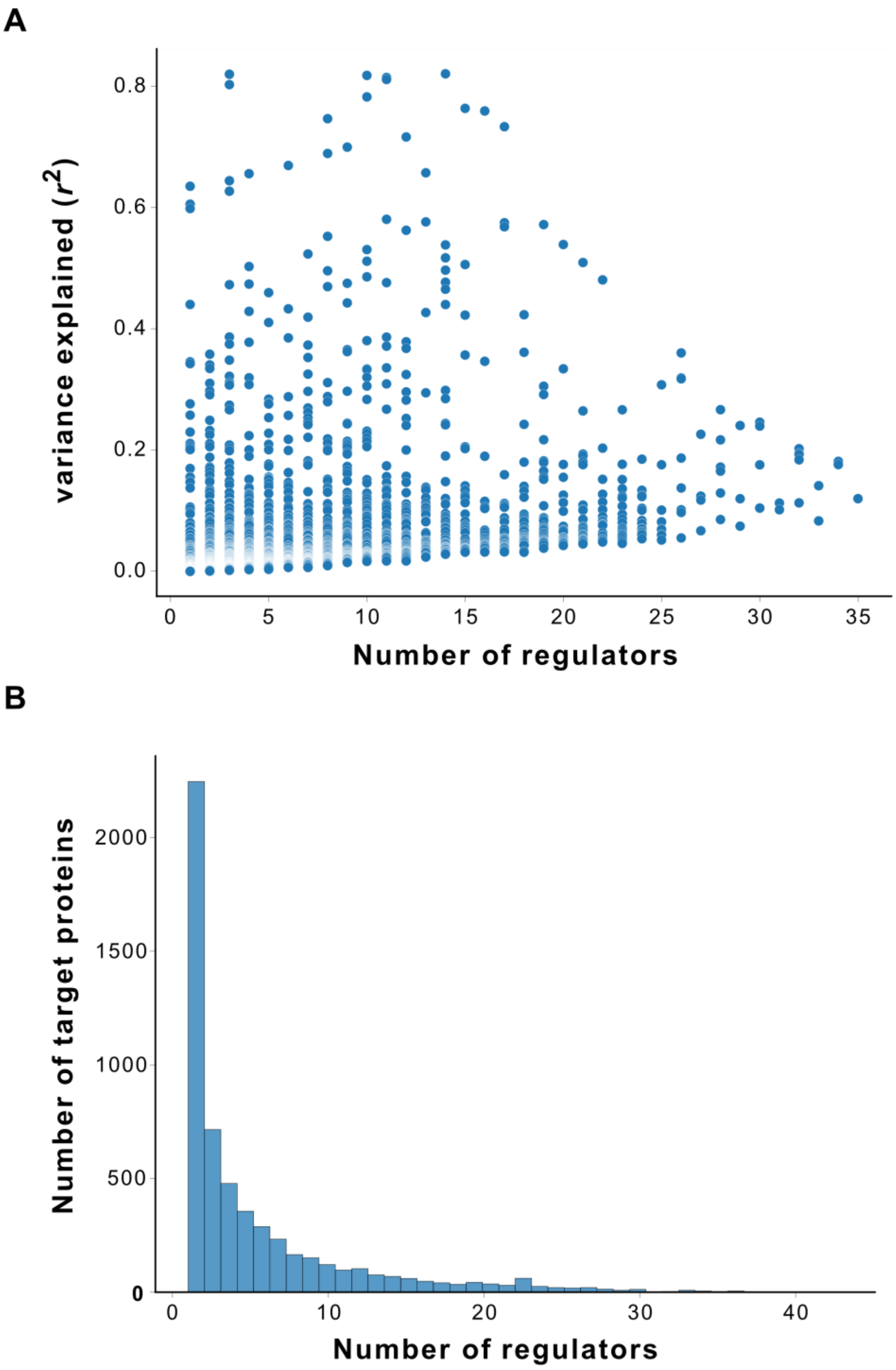

Supplementary Fig. S3. **A**. The proportion of variance in protein expression (adjusted r^2) explained by *cis*-acting pQTLs for the regulatory proteins' impact on 5,459 target proteins in the CPN. **B.** The number of network regulators each of the 5,499 target proteins has.

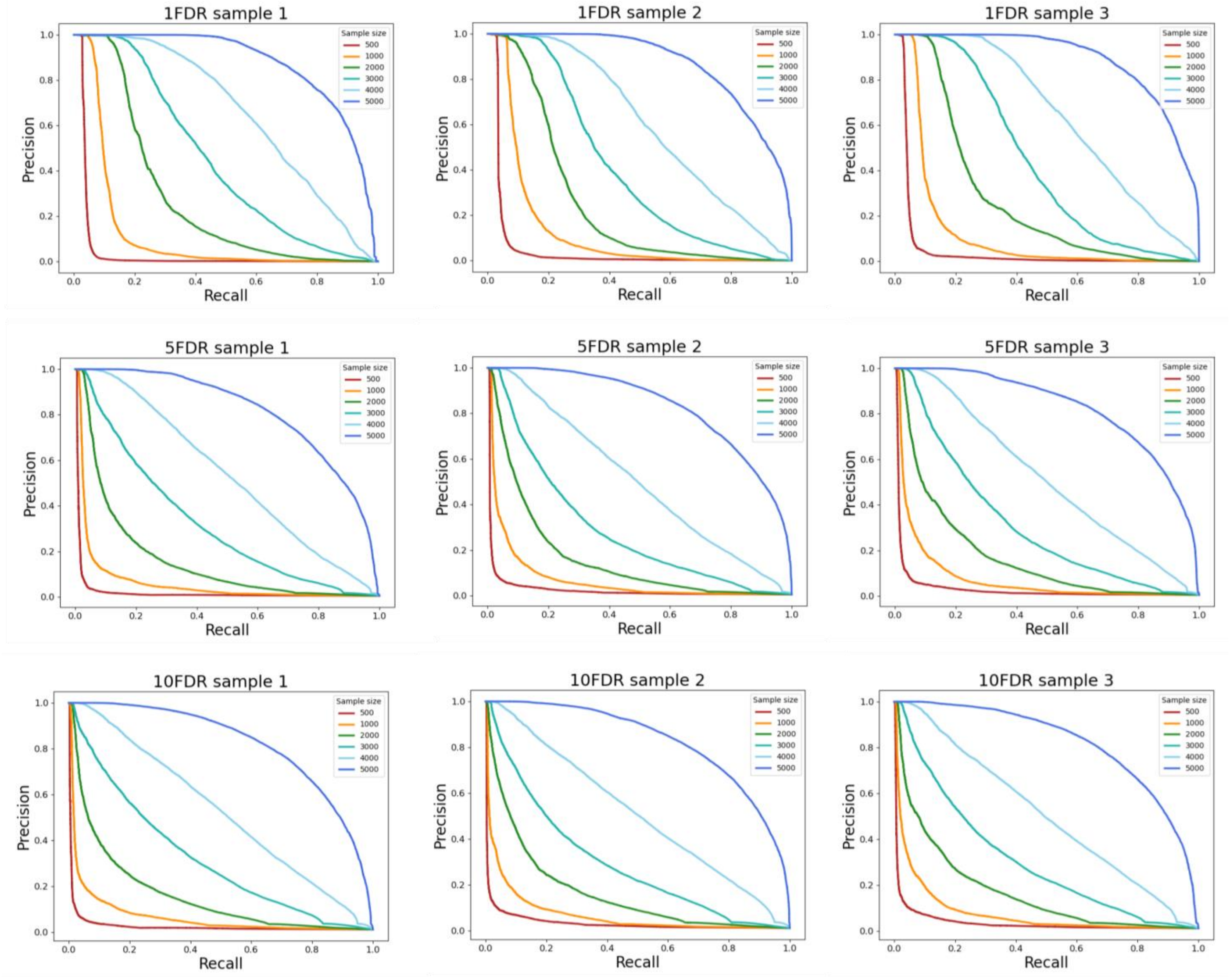

Supplementary Fig. S4. Precision-Recall curves were generated for sub-sampled networks and compared to networks using the full set of samples. Three random sub-samples of AGES participants were selected at different thresholds: 500, 1000, 2000, 3000, 4000, and 5000 samples, and networks reconstructed for each of these sub-sample test sets. The network generated from the full sample set served as the ground truth, filtered at 5%, and 10% FDR. These ground truth networks were represented as flattened matrices (i,j), where i = network regulator and j = target, with a value of 1 indicating an edge and 0 indicating no edge. Receiver operating characteristic (ROC) AUC and precision-recall were calculated for each sub-sampled network and compared against the ground truth networks.

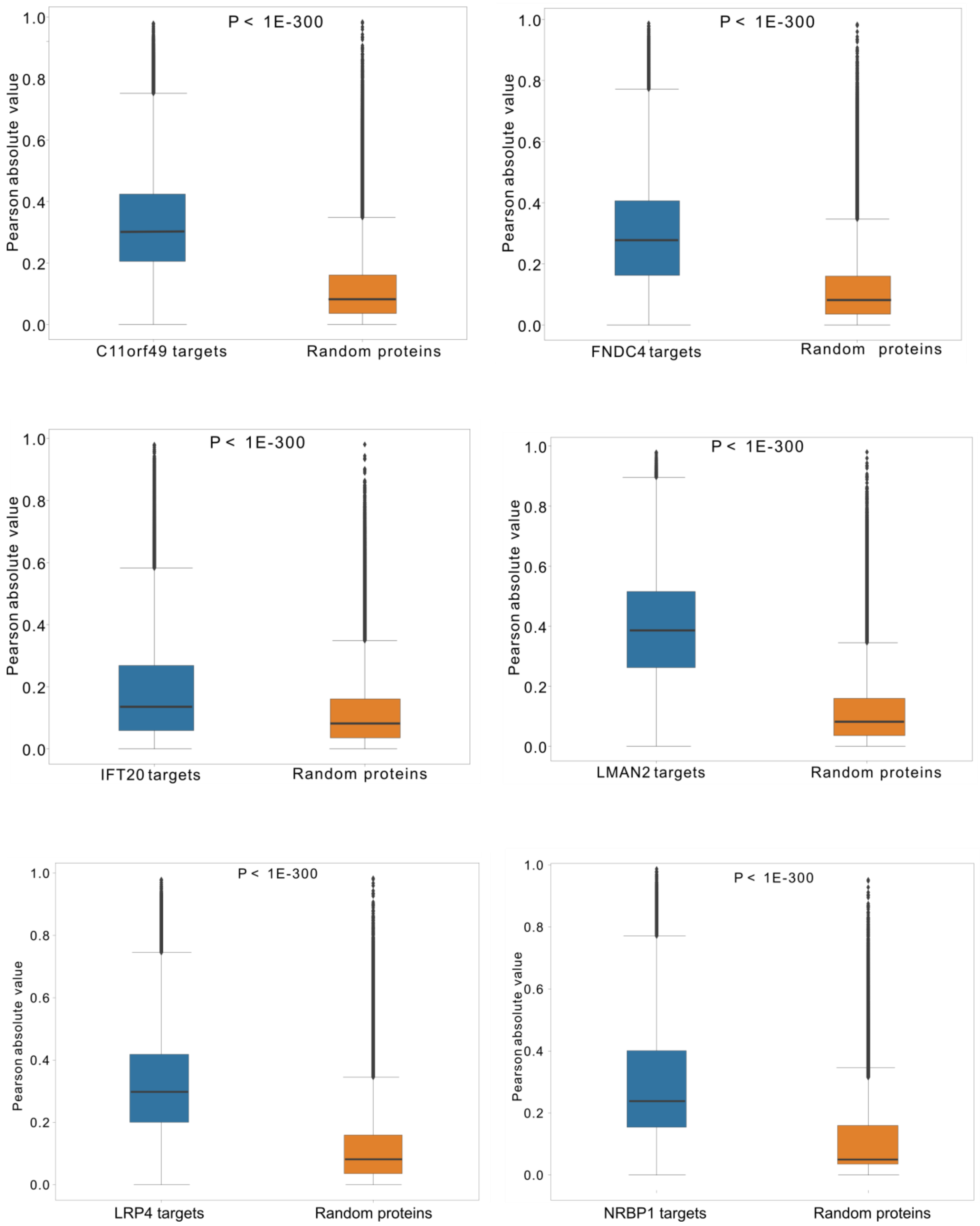
P < 1E-300
Pearson absolute value
C11orf49 targets
Random proteins
P < 1E-300
Pearson absolute value
FNDC4 targets
Random proteins
P < 1E-300
Pearson absolute value
IFT20 targets
Random proteins
P < 1E-300
Pearson absolute value
LMAN2 targets
Random proteins
P < 1E-300
Pearson absolute value
LRP4 targets
Random proteins
P < 1E-300
Pearson absolute value
NRBP1 targets
Random proteins

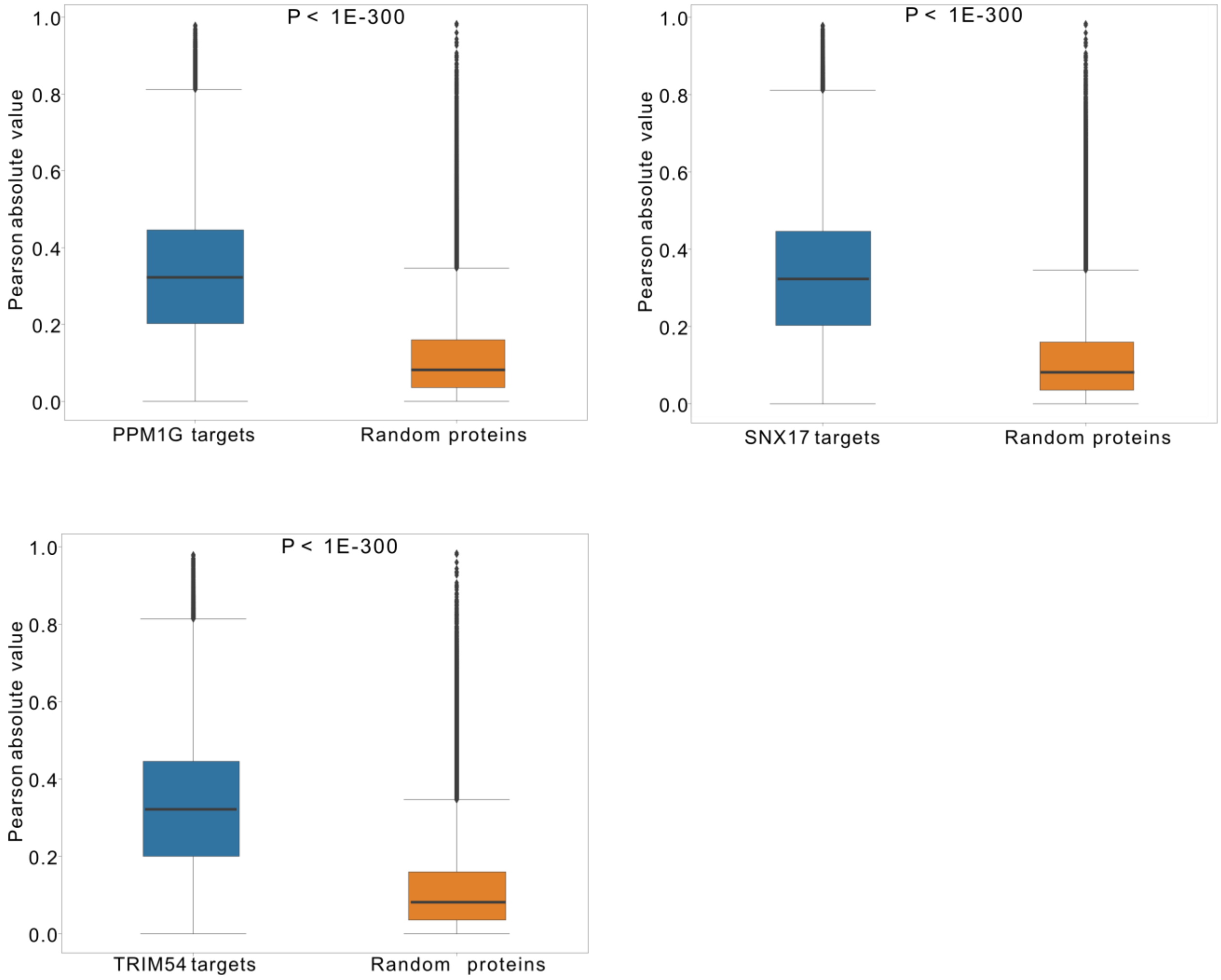

Supplementary Fig. S5. Box plots of a pairwise Pearson correlations between all proteins in a given CPN (blue boxes) compared to pairwise correlations between random proteins of the same size (yellow brown boxes). The P-value is a Kruskal Wallis test for the predicted vs random distributions.

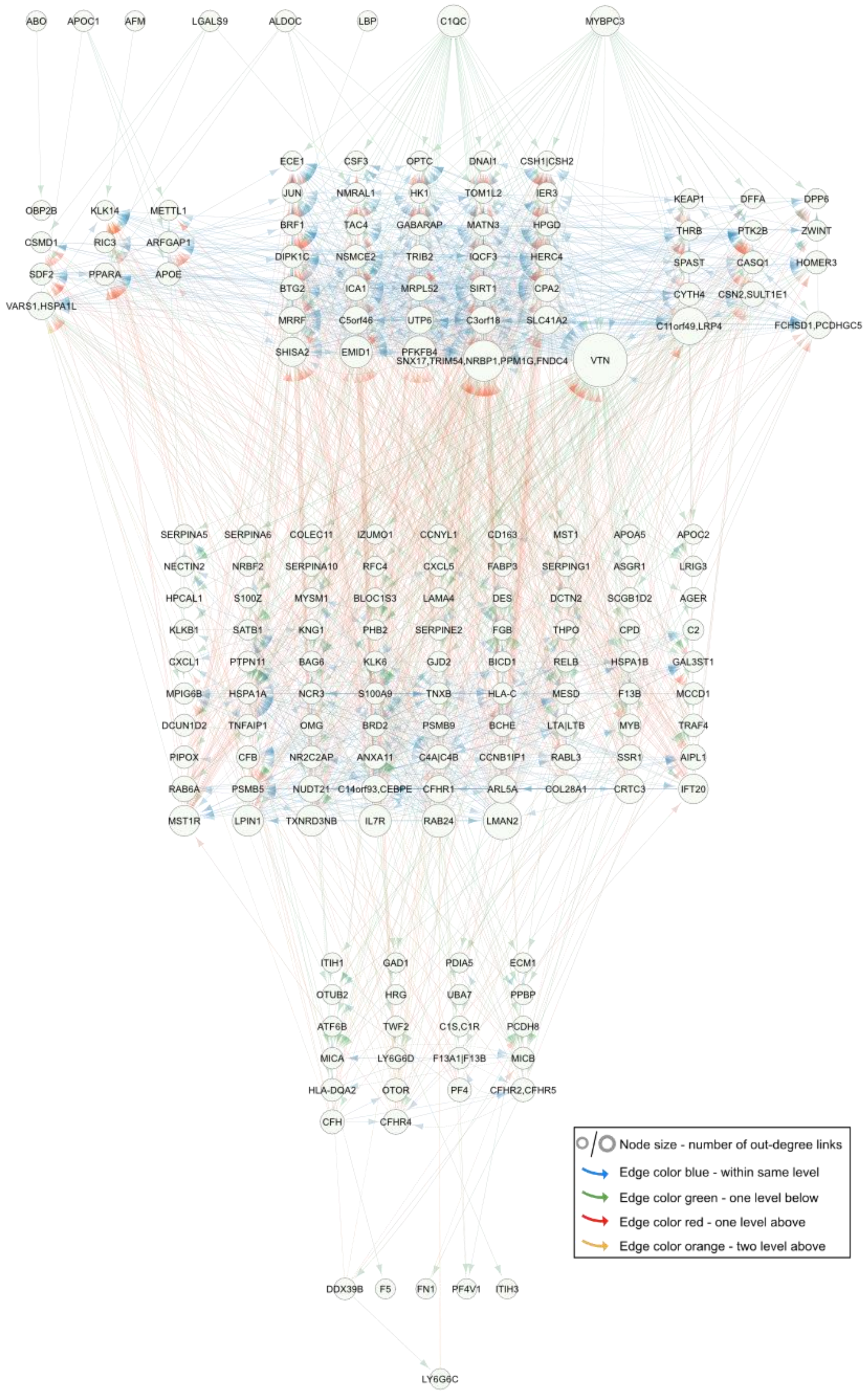

Supplementary Fig. S6. Network visualization of causal interactions among the 185 CPNs with more than 10 targets (FDR = 1%), with no edges removed. Refer to the comparison with the DAG Bayesian network in Fig. 3 of the main text.

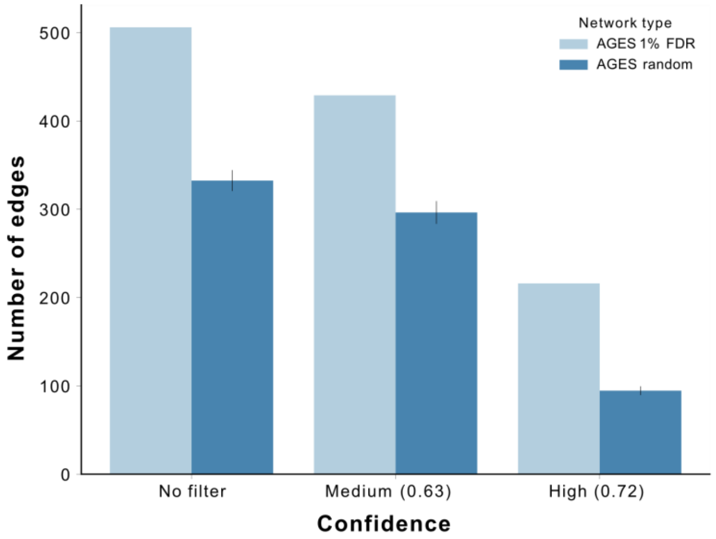

Supplementary Fig. S7. The CPN networks identified in the AGES study were compared against 289,112 protein-protein interactions (PPIs) sourced from the human integrated protein-protein interactions reference database at varying confidence thresholds (see Methods).

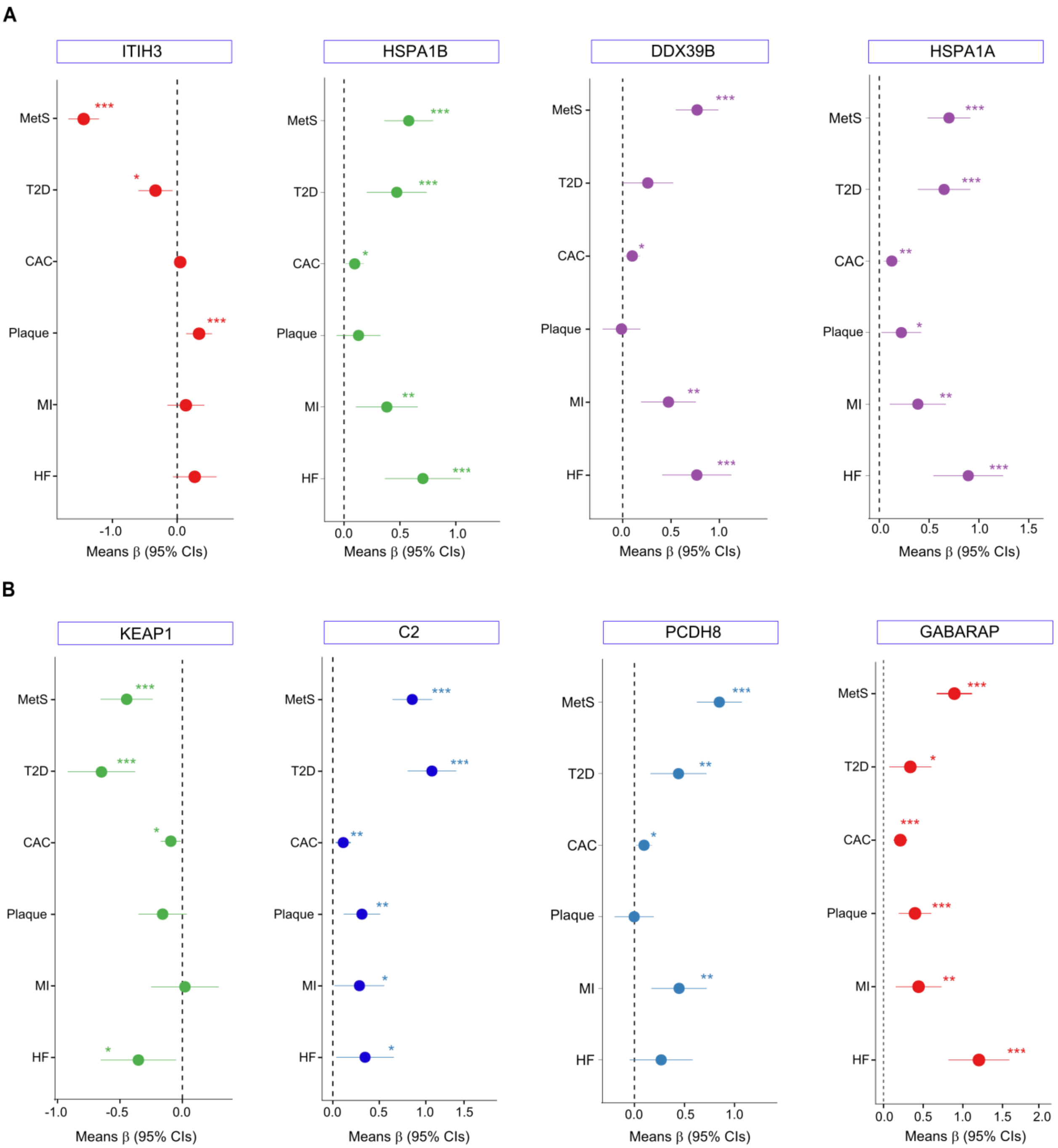
A
ITIH3
HSPA1B
DDX39B
HSPA1A
MetS
T2D
CAC
Plaque
MI
HF
Means β (95% CIs)
B
KEAP1
C2
PCDH8
GABARAP

C

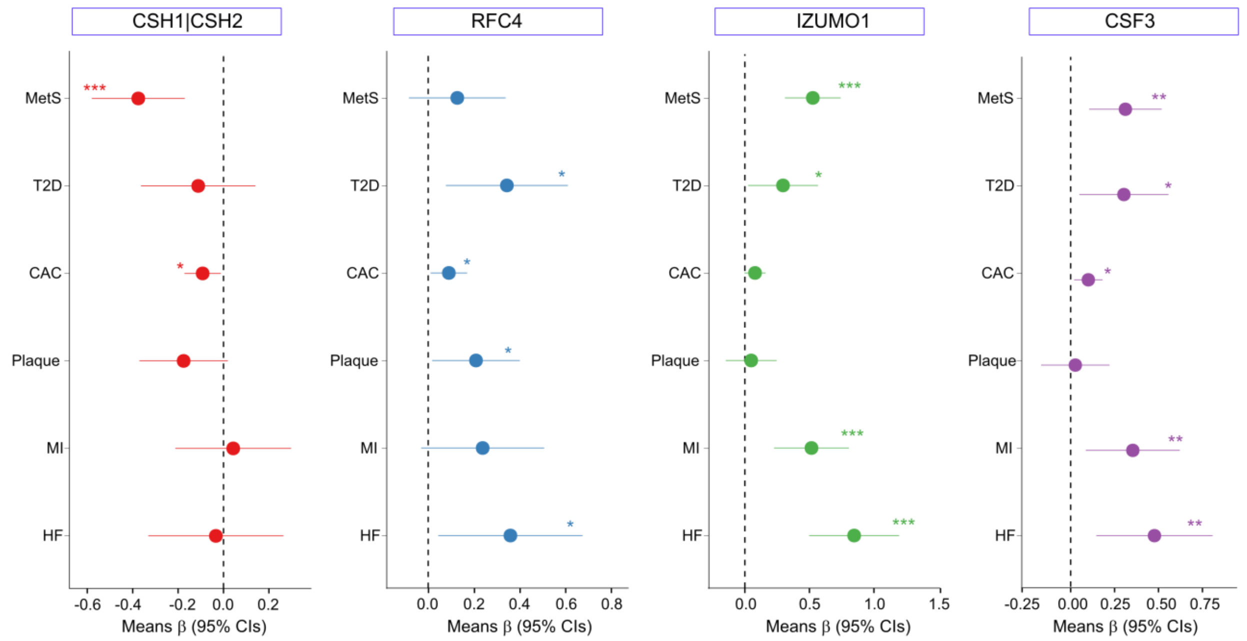
CSH1|CSH2
RFC4
IZUMO1
CSF3
MetS
T2D
CAC
Plaque
MI
HF
-0.6 -0.4 -0.2 0.0 0.2
0.0 0.2 0.4 0.6 0.8
0.0 0.5 1.0 1.5
-0.25 0.00 0.25 0.50 0.75
Means β (95% CIs)

D

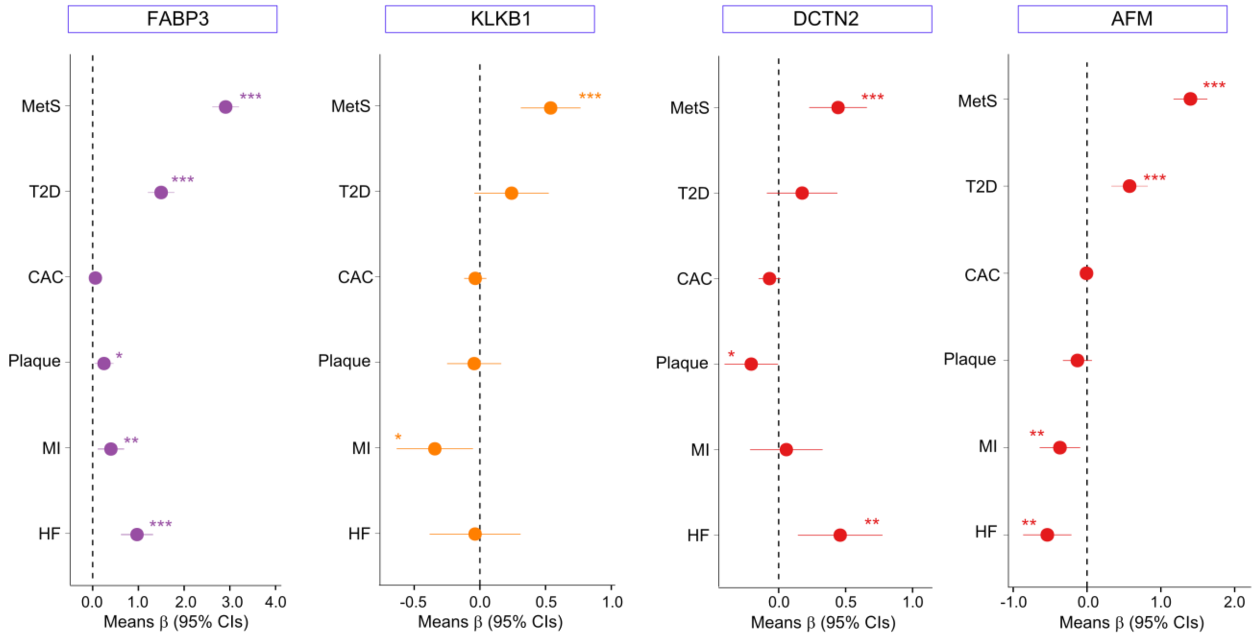
FABP3
KLKB1
DCTN2
AFM
MetS
T2D
CAC
Plaque
MI
HF
0.0 1.0 2.0 3.0 4.0
-0.5 0.0 0.5 1.0
0.0 0.5 1.0
-1.0 0.0 1.0 2.0
Means β (95% CIs)

E

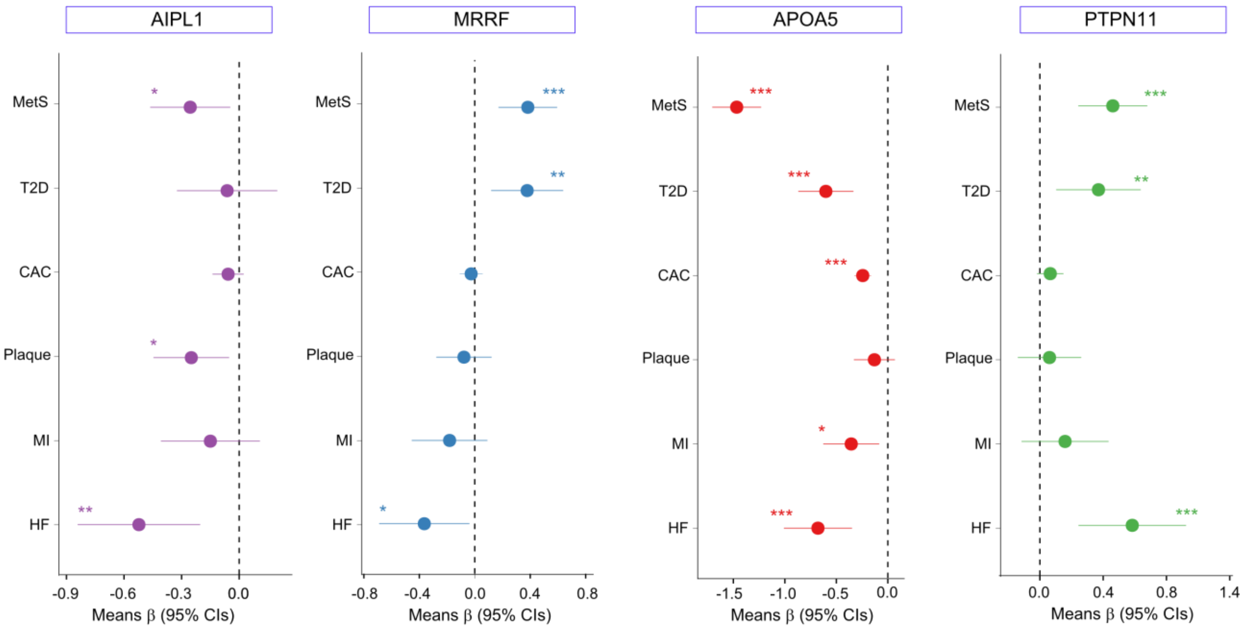
AIPL1
MRRF
APOA5
PTPN11
MetS
T2D
CAC
Plaque
MI
HF
-0.9 -0.6 -0.3 0.0
-0.8 -0.4 0.0 0.4 0.8
-1.5 -1.0 -0.5 0.0
0.0 0.4 0.8 1.4
Means β (95% CIs)

F

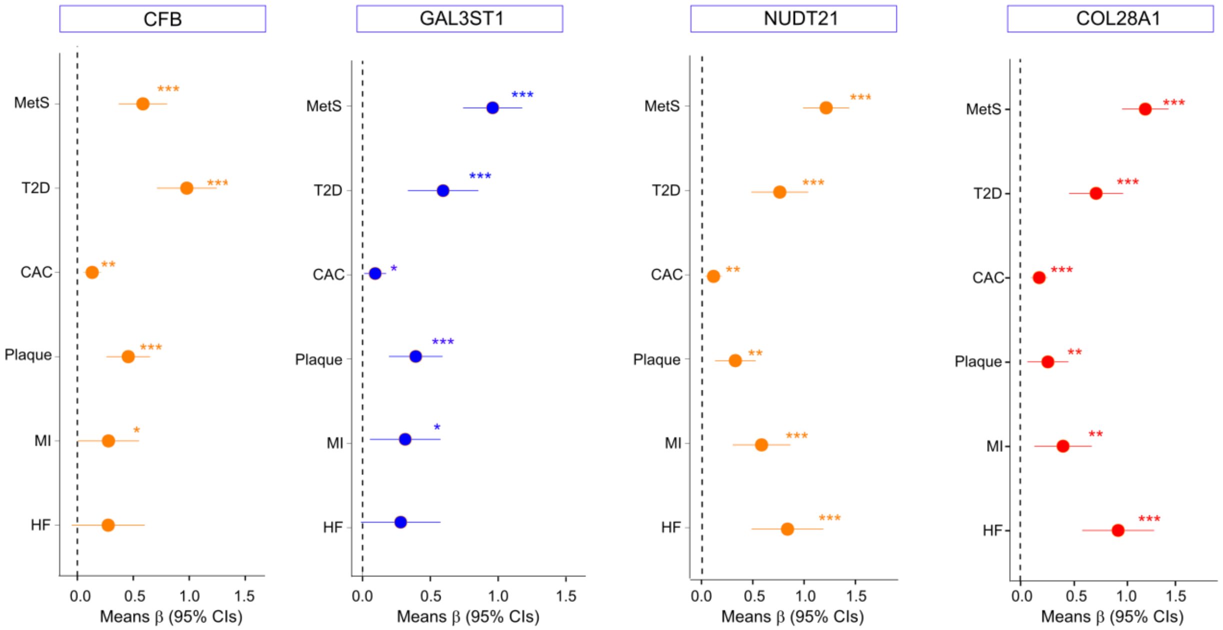
CFB
GAL3ST1
NUDT21
COL28A1
MetS
T2D
CAC
Plaque
MI
HF
0.0 0.5 1.0 1.5
Means β (95% CIs)

G

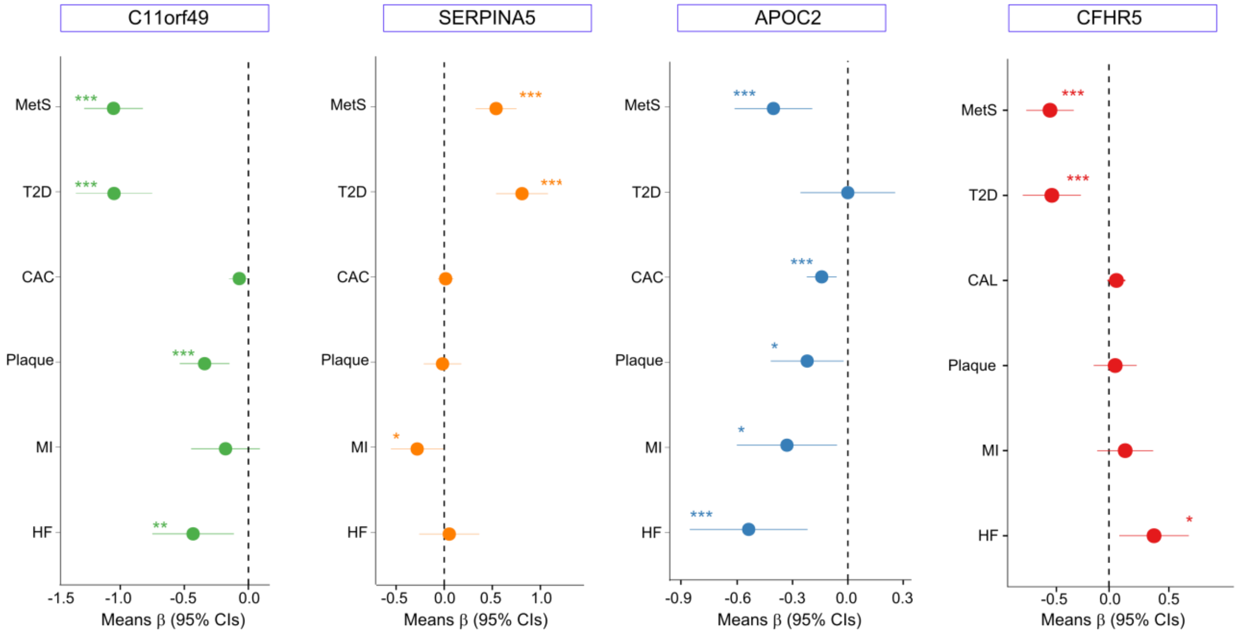
C11orf49
SERPINA5
APOC2
CFHR5
MetS
T2D
CAC
CAL
Plaque
MI
HF
Means β (95% CIs)

H

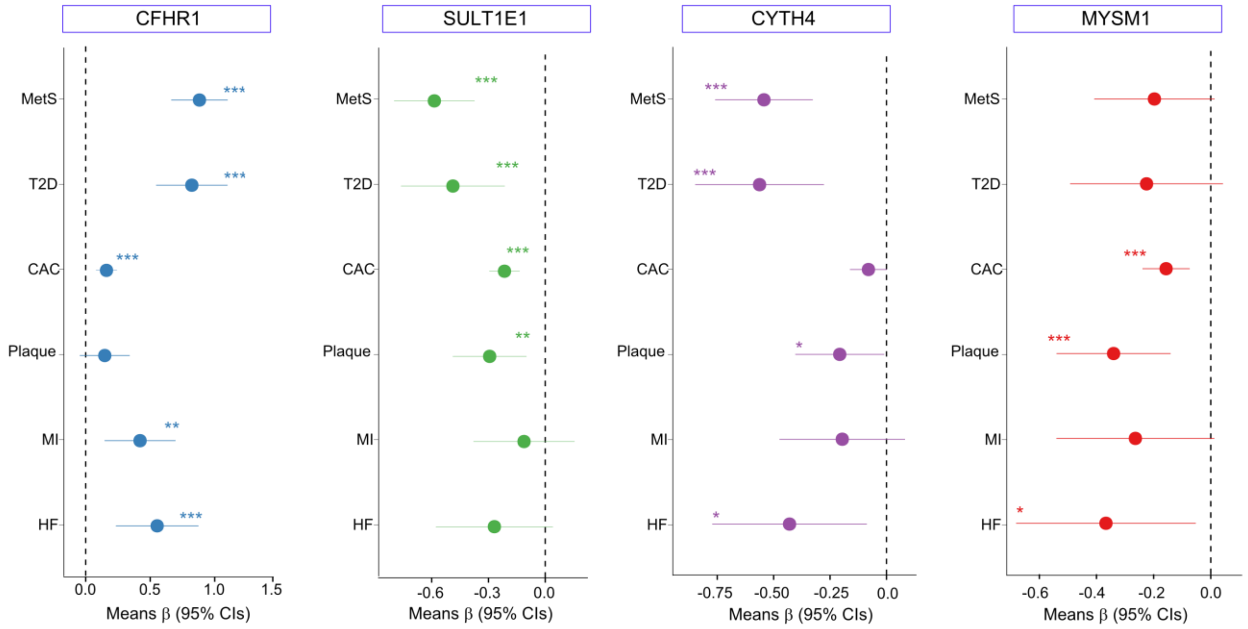
CFHR1
SULT1E1
CYTH4
MYSM1
MetS
T2D
CAC
Plaque
MI
HF
Means β (95% CIs)

I

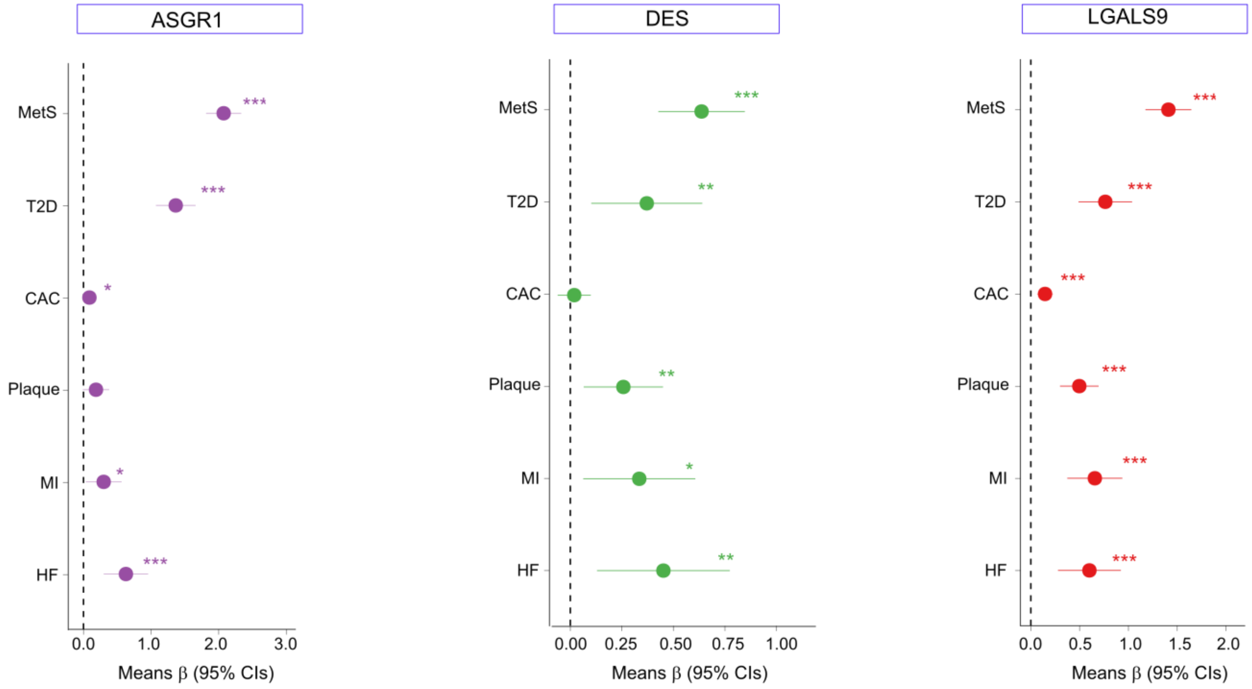

Supplementary Fig. S8. The difference in various outcome values between the fifth (top) and first (bottom) quintiles of the network regulators (**A-I**) from the top ranking CPNs. For each aptamer, we split the AGES cohort into quintile groups and calculate associations between the group (treated as a factor variable) and an assortment of outcomes. In the case of a continuous outcome, we use ordinary regression, and in the case of binary outcomes we use logistic regression for prevalent disease and Cox proportional-hazards model fitted to the box cox transformed serum proteomics data. All statistical results are obtained using linear models in the case of continuous outcomes and generalized linear models for binary outcomes. The models were fit using the outcomes as dependent variables and protein quintiles as predictor variables along with any adjustment variables including age or sex. The protein quintiles are treated as factor variables so there are no underlying assumptions regarding linear effects or other functional forms. Continuous outcomes are standardized prior to model fitting so coefficient estimates should be interpreted on the standard deviation scale, i.e. an estimated mean difference of 1 between protein quintiles translates to a one-standard-deviation difference between groups after adjusting for other included variables. The expected means are obtained as linear predictions from the fitted models along with the fitted confidence intervals around the mean. The linear predictions for qualitative phenotypes are shown on the log-odds scale. The difference between the 5th and 1st protein quintiles (or any other quintiles) is obtained as the expected marginal difference between those groups after adjusting for any other included variables with unadjusted p-values. As such, for continuous outcomes they are the optimal linear estimator with corresponding confidence intervals and p-values, but for discrete outcomes they are obtained using commonly applied asymptotic approximations. MetS, metabolic syndrome; T2D, type two diabetes; CAC, coronary artery calcium; Plaque, carotid plaque severity score; MI, incident myocardial infarction; HF, incident heart failure. ***(P-value < 0.001, two-sided), **(P-value < 0.01, two-sided), *(P-value < 0.05, two-sided).

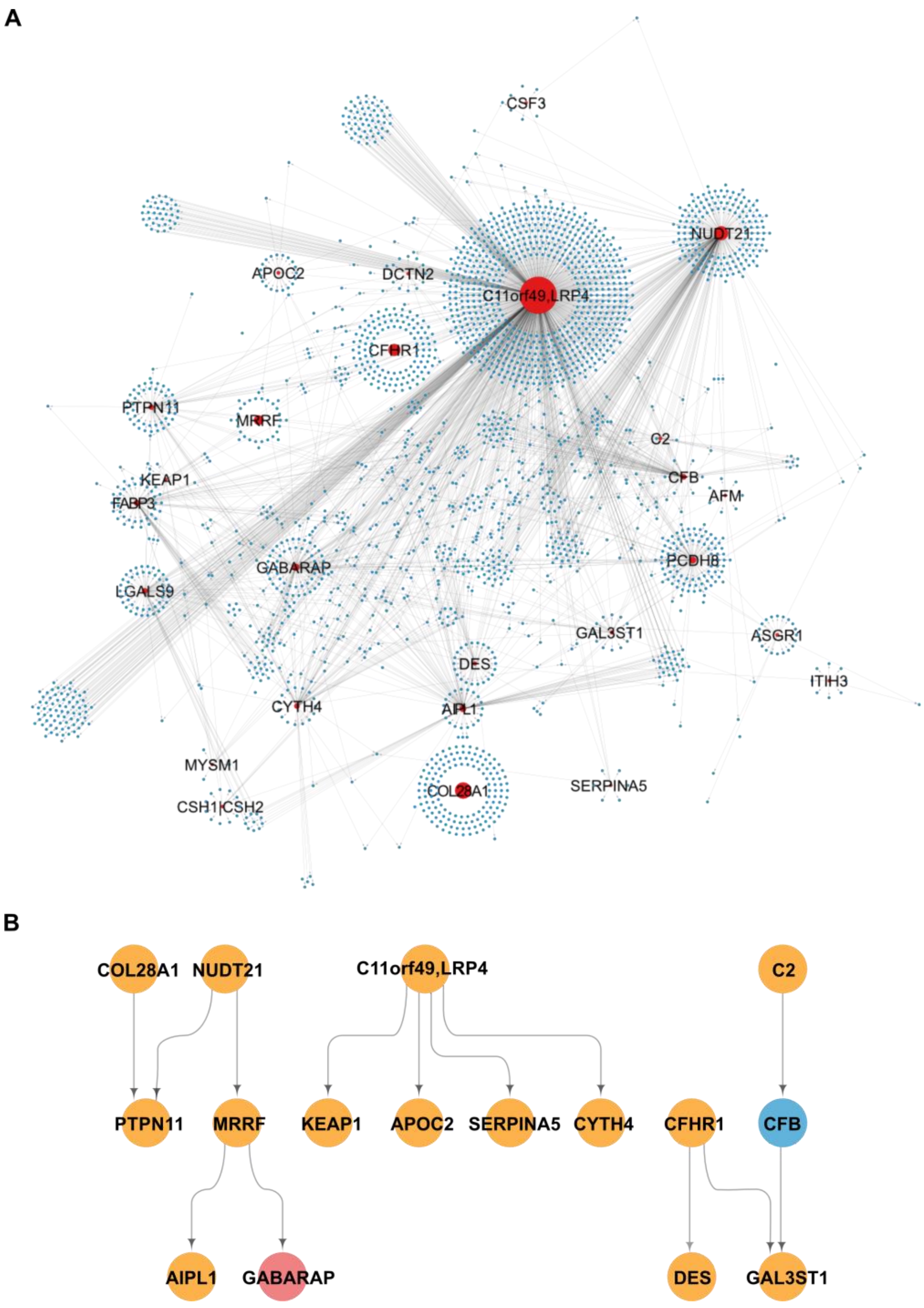

Supplementary Fig. S9. **A**. Network visualization of top ranked CPNs linked to MI-linked traits (arbitrary ranking score > 6) where network eigen-protein variance explained > 30%. Red nodes represent regulatory A-proteins and blue nodes represent target B-proteins. **B**. A hierarchical representation of regulatory A-proteins, where color indicates the degree of association with incident MI. Blue signifies no association, yellow indicates that either the eigen-protein or A-protein is associated, and red denotes that both the eigen-protein and A-protein are associated. The eigen-protein PC1 was required to explain at least 30% of the variance (refer to the top-ranked networks for incident MI and HF related traits in Supplementary Data 7-8).

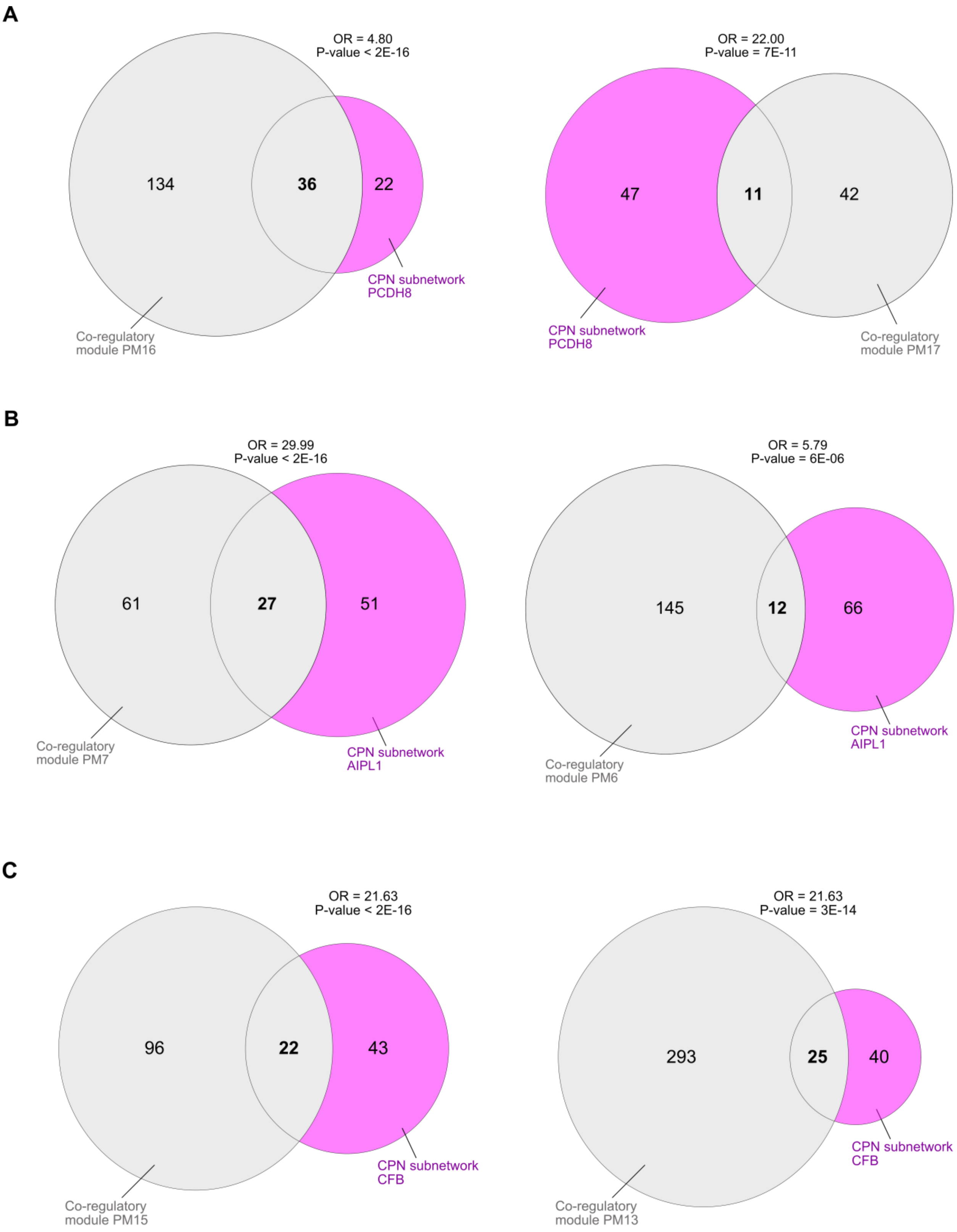
A
OR = 4.80
P-value < 2E-16
134
36
22
CPN subnetwork
PCDH8
Co-regulatory
module PM16
OR = 22.00
P-value = 7E-11
47
11
42
CPN subnetwork
PCDH8
Co-regulatory
module PM17
B
OR = 29.99
P-value < 2E-16
61
27
51
Co-regulatory
module PM7
CPN subnetwork
AIPL1
OR = 5.79
P-value = 6E-06
145
12
66
CPN subnetwork
AIPL1
Co-regulatory
module PM6
C
OR = 21.63
P-value < 2E-16
96
22
43
CPN subnetwork
CFB
Co-regulatory
module PM15
OR = 21.63
P-value = 3E-14
293
25
40
CPN subnetwork
CFB
Co-regulatory
module PM13

D

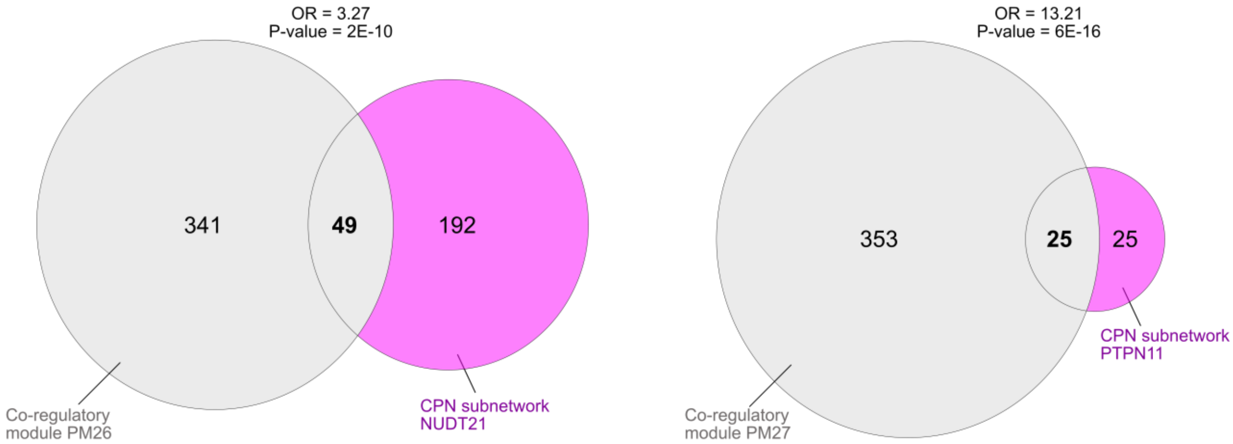

Supplementary Fig. S10. Examples (**A**-**D**) of Fisher's exact test results for the overlap between top-ranked circulating causal protein networks (CPN) in Table 2 of the main text, and the serum protein co-regulatory networks (PM) from Emilsson et al.[2]. For this comparison we focused solely on the proteins detected by both the 5K and 7K aptamer platforms. Complete details of these overlaps can be found in Supplementary Data 9.

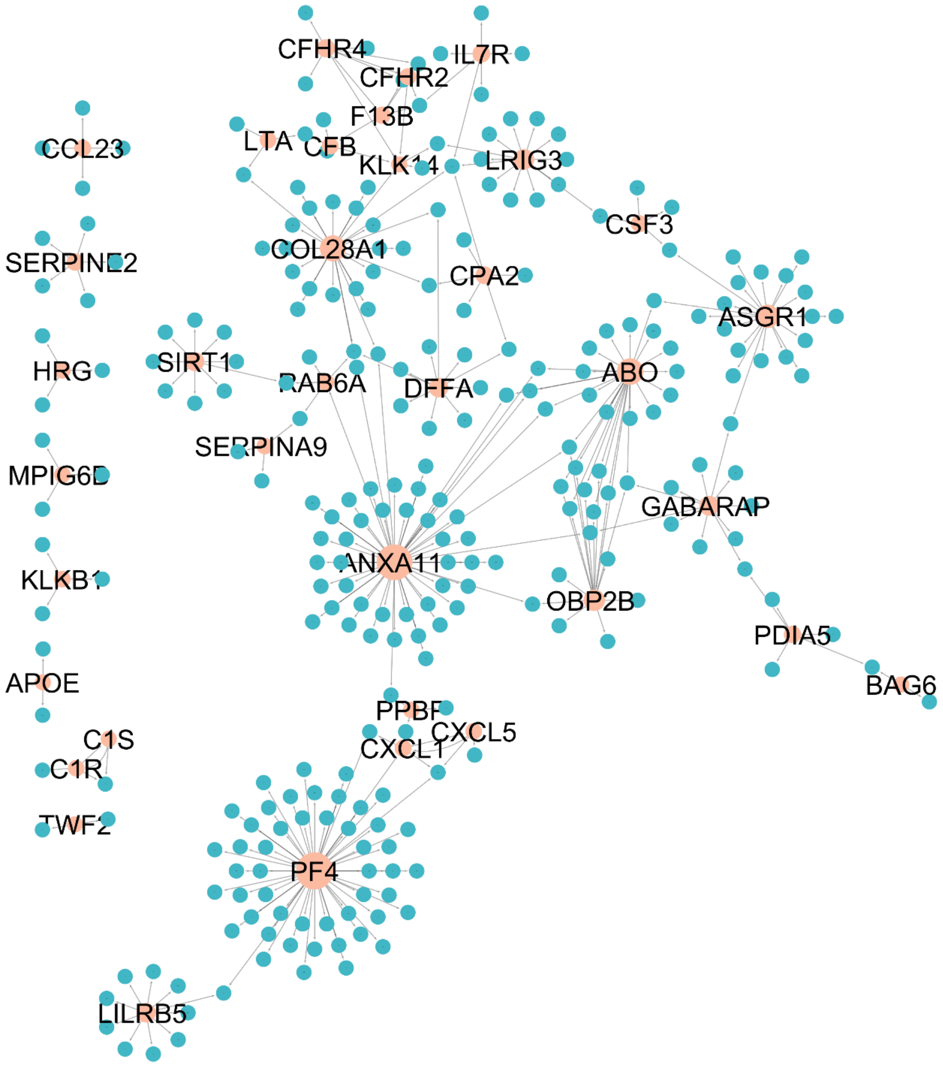

Supplementary Fig. S11. Network visualization from the comparative CPN analysis between AGES and UK Biobank. Edges visualized were identified in both CPNs (FDR = 1%) and filtered to remove network regulators with single targets. 36 network regulators are labelled and shown in red and there are 281 unique target proteins shown in blue (some red regulators may also be targets).

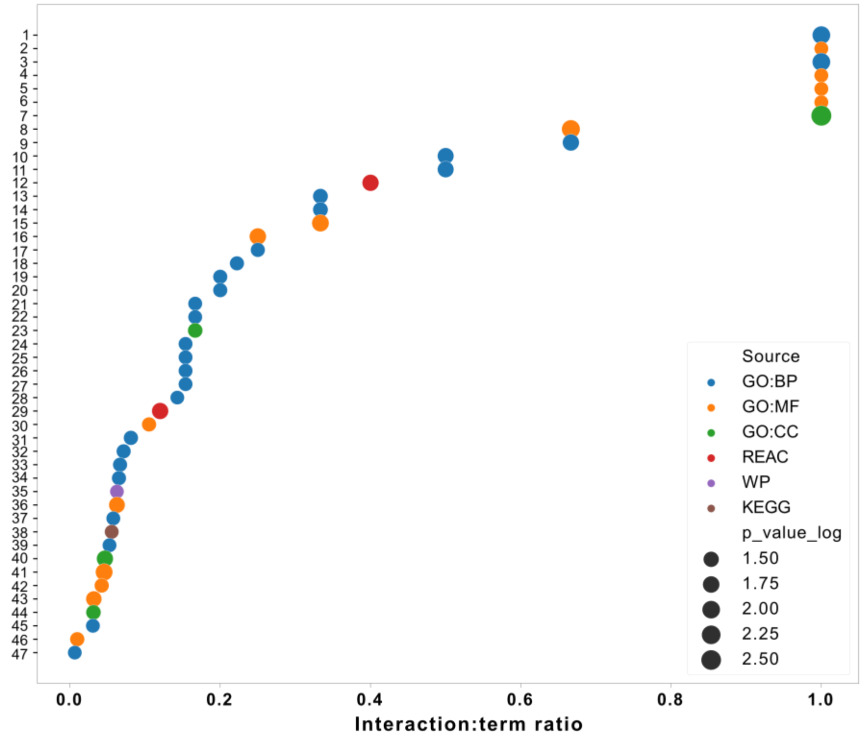

1. Cellular heat acclimation
2. Granulocyte colony-stimulating factor receptor binding
3. Heat acclimation
4. Farnesylated protein binding
5. Denatured protein binding
6. Vitamin E binding
7. Classical-complement-pathway C3/C5 convertase complex
8. C3HC4-type RING finger domain binding
9. Positive regulation of microtubule nucleation
10. Positive regulation of nucleotide-binding oligomerization domain containing 2 signaling pathway
11. Positive regulation of nucleotide-binding domain, leucine rich repeat containing receptor signaling pathway
12. Activation of C3 and C5
13. Regulation of nucleotide-binding oligomerization domain containing 2 signaling pathway
14. Regulation of microtubule nucleation
15. ATP-dependent protein disaggregase activity
16. Protein-containing complex destabilizing activity
17. Regulation of nucleotide-binding domain, leucine rich repeat containing receptor signaling pathway
18. Positive regulation of tumor necrosis factor-mediated signaling pathway
19. Negative regulation of inclusion body assembly
20. Nucleotide-binding oligomerization domain containing 2 signaling pathway
21. Regulation of mitotic spindle assembly
22. Regulation of spindle assembly
23. Aggresome
24. Regulation of Rac protein signal transduction
25. Microtubule nucleation
26. Nucleotide-binding domain, leucine rich repeat containing receptor signaling pathway
27. Positive regulation of microtubule polymerization
28. Regulation of inclusion body assembly
29. HSP90 chaperone cycle for steroid hormone receptors (SHR) in the presence of ligand
30. ATP-dependent protein folding chaperone
31. Positive regulation of proteasomal ubiquitin-dependent protein catabolic process
32. Positive regulation of protein polymerization
33. Positive regulation of ubiquitin-dependent protein catabolic process
34. Positive regulation of proteasomal protein catabolic process
35. Complement and coagulation cascades
36. Histone deacetylase binding
37. Mitotic spindle organization
38. Spliceosome
39. Positive regulation of proteolysis involved in protein catabolic process
40. Blood microparticle
41. ATP hydrolysis activity
42. Unfolded protein binding
43. ATP-dependent activity
44. Nuclear speck
45. Glycerolipid metabolic process
46. Enzyme binding
47. Cellular component organization

Supplementary Fig. S12. A functional enrichment analysis conducted on the 25 network regulators from the top ranking CPNs. The bubble plot displays the ratio of interaction size to term size, with the dot size representing the -log10 P-value. Significant enrichment was defined as FDR < 5%.

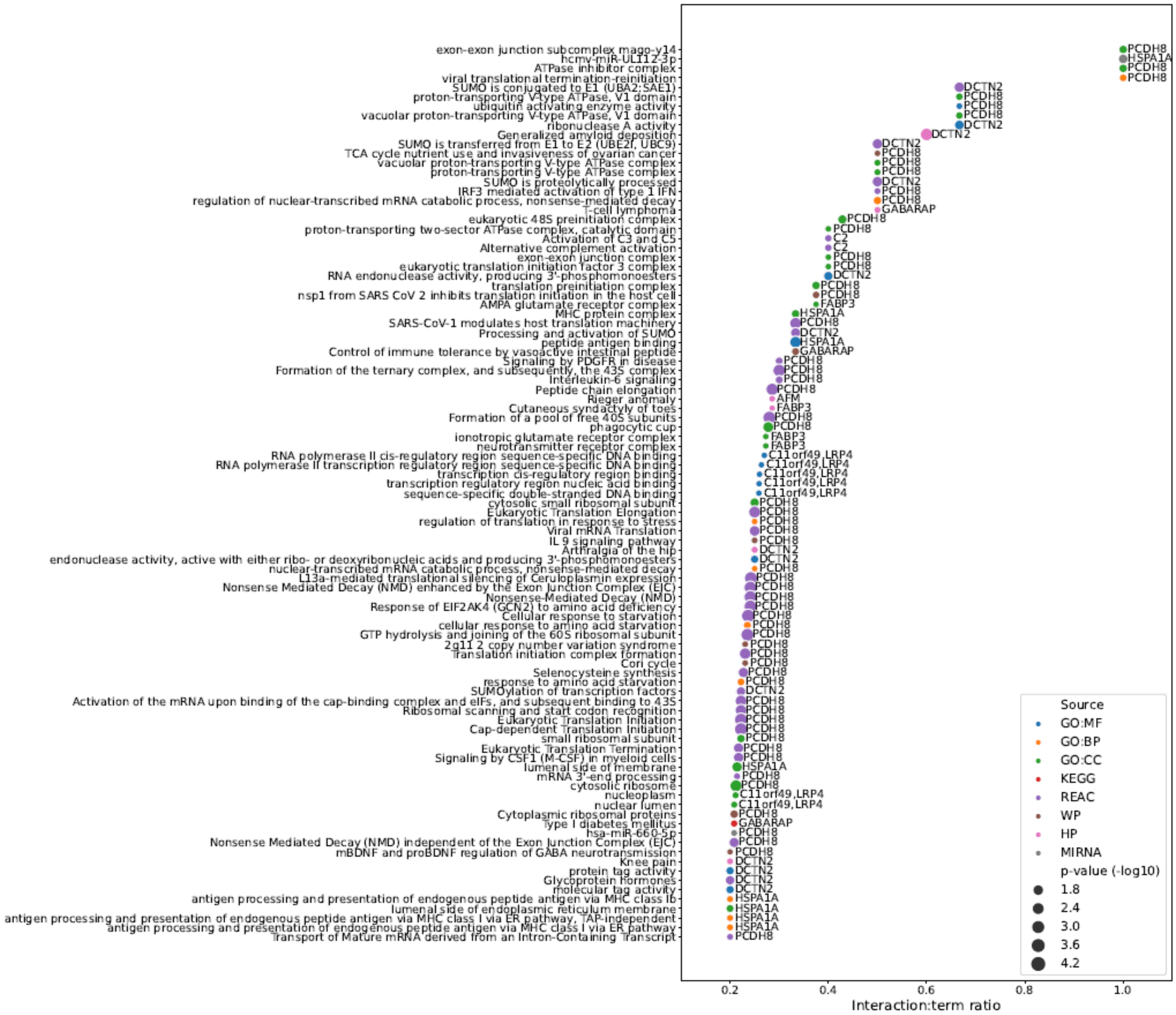

Supplementary Fig. S13. GO enrichments for all targets within each top-ranked CPN subnetwork were analyzed using g:Profiler, like that in Figure S12, with the respective networks labeled on the plot. These enrichments have been filtered to an FDR < 5% and an interaction: term size ratio of > 0.2. A comparable enrichment analysis for the corresponding target proteins is provided in Supplementary Data 11.

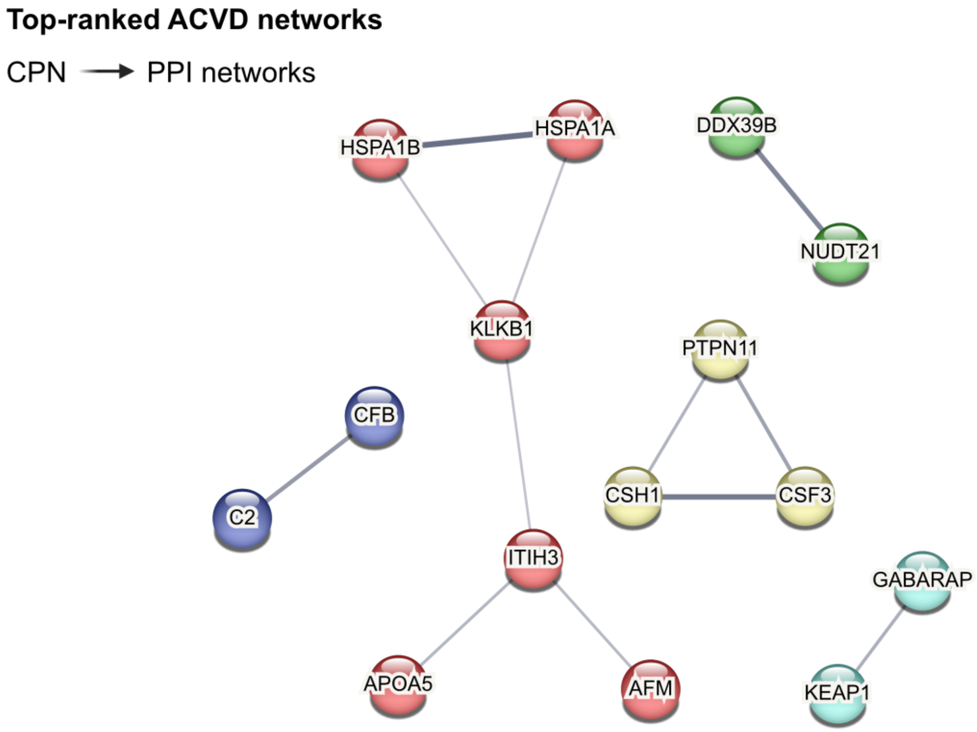

Supplementary Fig. S14. Protein-protein interactions among network regulators within the top-ranked CPN subnetworks, as identified by the STRING database[9]. These edges represent functional and physical interactions, and unconnected network regulators are excluded from the visualization.

Supplementary References

1. Alanis-Lobato, G., Andrade-Navarro, M.A. & Schaefer, M.H. HIPPIE v2.0: enhancing meaningfulness and reliability of protein-protein interaction networks. *Nucleic Acids Res* **45**, D408-d414 (2017).
2. Emilsson, V., *et al.* Co-regulatory networks of human serum proteins link genetics to disease. *Science* **361**, 769-773 (2018).
3. Emilsson, V., *et al.* A proteogenomic signature of age-related macular degeneration in blood. *Nat Commun* **13**, 3401 (2022).
4. Mreisat, A., Kanaani, H., Saada, A. & Horowitz, M. Heat acclimation mediated cardioprotection is controlled by mitochondrial metabolic remodeling involving HIF-1α. *Journal of thermal biology* **93**, 102691 (2020).
5. Harada, M., *et al.* G-CSF prevents cardiac remodeling after myocardial infarction by activating the Jak-Stat pathway in cardiomyocytes. *Nat Med* **11**, 305-311 (2005).
6. Worman, H.J. & Michaelis, S. Permanently Farnesylated Prelamin A, Progeria, and Atherosclerosis. *Circulation* **138**, 283-286 (2018).
7. Ziegler, M., Wallert, M., Lorkowski, S. & Peter, K. Cardiovascular and Metabolic Protection by Vitamin E: A Matter of Treatment Strategy? *Antioxidants (Basel, Switzerland)* **9**(2020).
8. Kiss, M.G. & Binder, C.J. The multifaceted impact of complement on atherosclerosis. *Atherosclerosis* **351**, 29-40 (2022).
9. Szklarczyk, D., *et al.* STRING v11: protein-protein association networks with increased coverage, supporting functional discovery in genome-wide experimental datasets. *Nucleic Acids Res* **47**, D607-d613 (2019).
10. Ebana, Y., *et al.* A functional SNP in ITIH3 is associated with susceptibility to myocardial infarction. *Journal of human genetics* **52**, 220-229 (2007).
11. Roberts, J.A., Rainbow, R.D. & Sharma, P. Mitigation of Cardiovascular Disease and Toxicity through NRF2 Signalling. *International journal of molecular sciences* **24**(2023).
12. Zoccarato, A., *et al.* NRF2 activation in the heart induces glucose metabolic reprogramming and reduces cardiac dysfunction via upregulation of the pentose phosphate pathway. *Cardiovascular research* **121**, 339-352 (2025).
13. Graham, S.E., *et al.* The power of genetic diversity in genome-wide association studies of lipids. *Nature* **600**, 675-679 (2021).
14. Kurki, M.I., *et al.* FinnGen provides genetic insights from a well-phenotyped isolated population. *Nature* **613**, 508-518 (2023).
15. Jönsson, G., *et al.* Hereditary C2 deficiency in Sweden: frequent occurrence of invasive infection, atherosclerosis, and rheumatic disease. *Medicine (Baltimore)* **84**, 23-34 (2005).
16. Nelson, C.P., *et al.* Association analyses based on false discovery rate implicate new loci for coronary artery disease. *Nat Genet* **49**, 1385-1391 (2017).
17. Bortnick, A.E., *et al.* Plasma Proteomic Assessment of Calcific Aortic Valve Disease in Older Adults. *J Am Heart Assoc* **14**, e036336 (2025).
18. Lerman, D.A., Prasad, S. & Alotti, N. Calcific Aortic Valve Disease: Molecular Mechanisms and Therapeutic Approaches. *European cardiology* **10**, 108-112 (2015).
19. Pennacchio, L.A., *et al.* An apolipoprotein influencing triglycerides in humans and mice revealed by comparative sequencing. *Science* **294**, 169-173 (2001).
20. Buniello, A., *et al.* The NHGRI-EBI GWAS Catalog of published genome-wide association studies, targeted arrays and summary statistics 2019. *Nucleic Acids Res* **47**, D1005-d1012 (2019).

21. Araki, T., *et al.* Noonan syndrome cardiac defects are caused by PTPN11 acting in endocardium to enhance endocardial-mesenchymal transformation. *Proc Natl Acad Sci U S A* **106**, 4736-4741 (2009).
22. Schmidt, A.F., *et al.* Druggable proteins influencing cardiac structure and function: Implications for heart failure therapies and cancer cardiotoxicity. *Sci Adv* **9**, eadd4984 (2023).
23. Coan, P.M., *et al.* Complement Factor B Is a Determinant of Both Metabolic and Cardiovascular Features of Metabolic Syndrome. *Hypertension (Dallas, Tex. : 1979)* **70**, 624-633 (2017).
24. van der Harst, P. & Verweij, N. Identification of 64 Novel Genetic Loci Provides an Expanded View on the Genetic Architecture of Coronary Artery Disease. *Circ Res* **122**, 433-443 (2018).
25. Lindström, S., *et al.* Genomic and transcriptomic association studies identify 16 novel susceptibility loci for venous thromboembolism. *Blood* **134**, 1645-1657 (2019).
26. Yuan, S., *et al.* Plasma proteins and onset of type 2 diabetes and diabetic complications: Proteome-wide Mendelian randomization and colocalization analyses. *Cell reports. Medicine* **4**, 101174 (2023).
27. Dulin, E., García-Barreno, P. & Guisasola, M.C. Genetic variations of HSPA1A, the heat shock protein levels, and risk of atherosclerosis. *Cell stress & chaperones* **17**, 507-516 (2012).
28. Peters, A.E., *et al.* Proteomic Pathways across Ejection Fraction Spectrum in Heart Failure: an EXSCEL Substudy. *medRxiv* (2023).
29. Goel, H., *et al.* Heart-type fatty acid-binding protein: an overlooked cardiac biomarker. *Annals of medicine* **52**, 444-461 (2020).
30. Zhuang, L., *et al.* Fatty acid-binding protein 3 contributes to ischemic heart injury by regulating cardiac myocyte apoptosis and MAPK pathways. *American journal of physiology. Heart and circulatory physiology* **316**, H971-h984 (2019).
31. Szymura, S.J., *et al.* DDX39B interacts with the pattern recognition receptor pathway to inhibit NF-κB and sensitize to alkylating chemotherapy. *BMC biology* **18**, 32 (2020).
32. Matsumori, A. Nuclear Factor-κB is a Prime Candidate for the Diagnosis and Control of Inflammatory Cardiovascular Disease. *European cardiology* **18**, e40 (2023).
33. Liao, C.C., Xu, J.W., Huang, W.C., Chang, H.C. & Tung, Y.T. Plasma Proteomic Changes of Atherosclerosis after Exercise in ApoE Knockout Mice. *Biology* **11**(2022).
34. Hess, K., *et al.* Concurrent action of purifying selection and gene conversion results in extreme conservation of the major stress-inducible Hsp70 genes in mammals. *Sci Rep* **8**, 5082 (2018).
35. Wan, J., *et al.* Kallikrein augments the anticoagulant function of the protein C system in thrombin generation. *Journal of thrombosis and haemostasis : JTH* **20**, 48-57 (2022).
36. Kronenberg, F., *et al.* Plasma concentrations of afamin are associated with the prevalence and development of metabolic syndrome. *Circ Cardiovasc Genet* **7**, 822-829 (2014).
37. Nowicki, G.J., Ślusarska, B., Polak, M., Naylor, K. & Kocki, T. Relationship between Serum Kallistatin and Afamin and Anthropometric Factors Associated with Obesity and of Being Overweight in Patients after Myocardial Infarction and without Myocardial Infarction. *Journal of clinical medicine* **10**(2021).
