# Supplementary material for: Circulating causal protein networks linked to future risk of myocardial infarction
Source: Nat Commun. 2025 Dec 18;17:448. doi: 10.1038/s41467-025-67135-3 (PMC12800284; doi:10.1038/s41467-025-67135-3)
Supplement: Supplementary file 2 — Description of Additional Supplementary File [file 41467_2025_67135_MOESM2_ESM.pdf]

Description of Additional Supplementary Files

Supplementary Data 1: The protein members of the 185 causal protein subnetworks identified in the AGES study

Supplementary Data 2: Transitive reduction of the A-only directed acyclic graph

Supplementary Data 3: Findings on the association between MI-related phenotypes and network regulators. The association between serum protein levels and quantitative phenotypes was assessed using linear regression. For discrete traits, either logistic regression or Cox proportional-hazards models were employed. All regression analyses were adjusted for age and sex. Benjamini-Hochberg false discovery rate (FDR) estimates were used to determine statistical significance. Abbreviations: CAC, coronary artery calcium; Plaque severity, carotid plaque severity score; T2D, type 2 diabetes; MI, myocardial infarction; HF, heart failure

Supplementary Data 4: Findings on the association between MI-related phenotypes and network eigenproteins (1st principal component with variance ≥ 15%). The association between serum protein levels and quantitative phenotypes was assessed using linear regression. For discrete traits, either logistic regression or Cox proportional-hazards models were employed. All regression analyses were adjusted for age and sex. A Bonferroni-adjusted P-value <0.00027 (0.05/185) was used to assess the significance of the associations. Abbreviations: CAC, coronary artery calcium; Plaque severity, carotid plaque severity score; T2D, type 2 diabetes; MI, myocardial infarction; HF, heart failure

Supplementary Data 5: Rank-based listing of network regulators according to their associations with MI-related traits. Abbreviations: CAC, coronary artery calcium; Plaque severity, carotid plaque severity score; T2D, type 2 diabetes; MI, myocardial infarction; HF, heart failure

Supplementary Data 6: Rank-based listing of eigen-proteins (PC1 ≥ 15% variance) according to their associations with MI-related traits. Abbreviations: CAC, coronary artery calcium; Plaque severity, carotid plaque severity score; T2D, type 2 diabetes; MI, myocardial infarction; HF, heart failure

Supplementary Data 7: Rank-based listing of eigen-proteins (PC1 ≥30% variance) according to their associations with MI-related traits. Abbreviations: CAC, coronary artery calcium; Plaque severity, carotid plaque severity score; T2D, type 2 diabetes; MI, myocardial infarction; HF, heart failure

Supplementary Data 8: The top ranking (PC1 ≥ 30%, arbitrary score ≥ 6) CPNs related to incident MI and associated traits

Supplementary Data 9: Overlap between the top-ranked CPN and the co-regulatory networks from Emilsson et al. (Science, 2018). FET refers to Fisher's exact test. "Module unassignment" denotes proteins that do not belong to a co-regulatory module (Emilsson et al., Science, 2018). FET P-values are reported as two-sided. NS means not significant; N/A means not applicable

Supplementary Data 10: Overlap of resolved CPNs reconstructed in UK Biobank and AGES. A one-sided P-value from the hypergeometric test is reported, and multiple testing addressed using the Storey–Tibshirani procedure to estimate q-values. N/A, not applicable

Supplementary Data 11: Functional enrichment analysis of the top ranked CPN subnetworks for links to MI and related traits, using the g:Profiler

Supplementary Data 12: The GWAS studies providing summary statistics data for the colocalization and MR analyses

Supplementary Data 13: Results of the Mendelian Randomization (MR) and colocalization analyses for ten of the top-ranked network regulators for MI and related traits. GWLS denotes the generalized weighted least squares method. Two-sided P-values are reported, and results with a false discovery rate (FDR) < 0.05 were considered statistically significant. N/A, not available

Supplementary Data 14: Sensitivity analyses of the network regulators identified as causally related to ACVD-associated traits (see Table S13). Q refers to Cochran's Q statistic, which measures instrument heterogeneity. LD clumping < 0.10. All P-values reported are two-sided

Supplementary Data 15: Directional associations of network regulators with core traits and survival outcomes. Depending on the outcome type: continuous, binary, or time-to-event, data were analyzed using linear regression, logistic regression, or Cox proportional hazards models, respectively. Analyses distinguished between prevalent and incident data

Supplementary Data 16: Candidate drugs and druggability scores for the top ranked network regulators
